# Mechanical restraint in inpatient psychiatric settings: A systematic review of international prevalence, associations, outcomes, and reduction strategies

**DOI:** 10.1192/j.eurpsy.2025.2453

**Published:** 2025-04-25

**Authors:** Daniel Whiting, Alexandra Lewis, Kursoom Khan, Eddie Alder, Gill Gookey, John Tully

**Affiliations:** 1 University of Nottingham, Institute of Mental Health, Nottingham, UK; 2 Nottinghamshire Healthcare NHS Foundation Trust, Nottingham, UK; 3 West London NHS Trust, London, UK; 4 Health Innovation East Midlands, Nottingham, UK

**Keywords:** mechanical restraint, physical restraint, restrictive practice, violence

## Abstract

**Background:**

There is increasing emphasis on reducing the use and improving the safety of mechanical restraint (MR) in psychiatric settings, and on improving the quality of evidence for outcomes. To date, however, a systematic appraisal of evidence has been lacking.

**Methods:**

We included studies of adults (aged 18–65) admitted to inpatient psychiatric settings. We included primary randomised or observational studies from 1990 onwards that reported patterns of MR and/or outcomes associated with MR, and qualitative studies referring to an index admission or MR episode. We presented prevalence data only for studies from 2010 onwards. The risk of bias was assessed using an adapted checklist for randomised/observational studies and the Newcastle-Ottawa scale for interventional studies.

**Results:**

We included 83 articles on 73 studies from 1990–2022, from 22 countries. Twenty-six studies, from 11 countries, 2010 onwards, presented data from on proportions of patients/admissions affected by MR. There was wide variation in prevalence (<1–51%). This appeared to be mostly due to variations in standard protocols between countries and regions, which dictated use compared to other restrictive practices such as seclusion. Indications for MR were typically broad (violence/aggression, danger to self or property). The most consistently associated factors were the early phase of admission, male sex, and younger age. Ward and staff factors were inconsistently examined. There was limited reporting of patient experience or positive effects.

**Conclusions:**

MR remains widely practiced in psychiatric settings internationally, with considerable variation in rates, but few high-quality studies of outcomes. There was a notable lack of studies investigating different types of restraint, indications, clinical factors associated with use, the impact of ethnicity and language, and evidence for outcomes. Studies examining these factors are crucial areas for future research. In limiting the use of MR, some ward-level interventions show promise, however, wider contextual factors are often overlooked.

## Introduction

Restrictive or coercive practices are used to maintain staff and patient safety in psychiatric hospital settings under relevant legal frameworks, but must only be undertaken in a manner that is compliant with human rights. There is increasing emphasis on reducing the use of these practices, or, when they are unavoidable, ensuring they are implemented as safely and briefly as possible. Restrictive interventions for managing behavioural disturbance encompass seclusion, chemical restraint, manual restraint using holds, and mechanical restraint (MR). Here, we define MR as per the UK’s Mental Health Act 1983 Code of Practice, as *“a form of restrictive intervention which involves the use of a device to prevent, restrict or subdue movement of a person’s body, or part of the body, for the primary purpose of behavioural control.”*

Although some attempts have been made to standardise practices across regions, for example, in Europe [1], patterns of the different types of restrictive practice still vary substantially. In some countries, only certain approaches are used [2], or even legal. Opinions and attitudes of staff, different legislation, and hospital policies [2, 3] appear to play a greater role than empirical data. One systematic review highlighted wide variation in rates, indications, and outcomes of the use of seclusion between The Netherlands, Finland, and the USA [4]. Standard clinical practices in different countries suggest this is likely also the case for MR. For example, in the UK, the use of MR is usually confined to secure hospitals, most commonly high security hospitals, or during the transfer of patients between secure settings, whereas in some European contexts, it is more commonly used in general adult settings. However, national and international patterns of use, and associated outcomes, are not understood in detail. Addressing this deficit is important due to unique ethical and acceptability considerations associated with MR.

Previous syntheses of evidence for MR in psychiatric settings have been limited in scope. A 2006 review explored short-term management of violence in adult psychiatric settings and emergency departments [5], however, MR was not emphasised. A Cochrane review on seclusion and restraint in the context of serious mental illness, last updated in 2012, only considered randomised trials, and so was not able to include any studies [6]. Two further reviews of seclusion and restraint have included wider observational study designs. One [7] narrowly defined MR as the “restraining of a patient to a bed using belts or straps,” and included only studies comparing seclusion and restraint with quantitative measures. The other [4] focused on adverse physical and mental outcomes, but forensic populations were excluded.

Together, the existing evidence base offers some insights into the current use of MR within the context of restrictive practice internationally, but a systematic appraisal of indications, patterns of use, regional variation, and outcomes, specific to MR, has been lacking. The current review addresses these gaps, by 1) focusing on MR only, 2) including a broad range of study designs and outcomes, including qualitative studies, and 3) clarifying the degree of regional variation in use. We also considered studies that examined the impact of interventions to reduce the use of MR, or the repercussions of ceasing its use. In so doing, we aimed to provide a comprehensive overview of available evidence specifically for MR, to inform policy and practice regarding its use in restrictive practices, and to provide clearer targets for future clinical research.

## Methods

We used standard systematic review methodology, with some adaptations in line with recent guidance from the Cochrane Rapid Reviews Methods Group [8–10] for the benefits of rapid evidence synthesis (title/abstract screening and data extraction were undertaken by a single reviewer with 20% cross-check). The review was pre-registered on PROSPERO (CRD42023472271).

### Search strategy

We searched MEDLINE, Embase, and PsycInfo for English language studies from inception to 7 September 2023, using a search strategy developed with information specialists [11] (Supplementary 1). We did not apply date limits to our search but made the subsequent decision to exclude studies conducted pre-1990, as, in keeping with large-scale work highlighting changes in psychiatric morbidity and treatment internationally from 1990 [12, 13], it was agreed among the review team that studies undertaken earlier are unlikely to be representative of contemporary psychiatric settings. For a clinically meaningful comparison of contemporary practice in relation to restrictive practice internationally, in our synthesis, we presented data separately for a subgroup of studies reporting data from 2010 onwards, given that this decade was characterised by the introduction in Europe of specific universal initiatives, such as the “Safewards” model [14].

### Eligibility assessment

Included studies were of adults (aged 18–65) admitted to inpatient psychiatric settings. Studies in youth samples and old age psychiatry samples, in which demographics likely introduce further variation, were beyond the scope of the current review. No diagnostic exclusion criteria were applied. Psychiatric assessment units within general emergency departments were not considered for inclusion.

MR was defined as any form of restrictive intervention involving the use of a device to prevent, restrict or subdue movement of a person’s body, or part of the body, for the primary purpose of behavioural control. Studies that did not disaggregate findings between MR and other forms of restrictive practice such as manual restraint, or did not specifically define the restraint method used, were excluded. Studies reporting restraint for the purposes of nasogastric feeding in patients with an eating disorder, or examining the restraint of patients in general medical settings such as intensive care units, were not considered for inclusion as these represent distinct clinical scenarios.

No comparator intervention was required for inclusion, however, studies in which MR was compared with other forms of restrictive intervention in terms of frequency of use or reasoning were considered for inclusion. Any reported intended or unintended effect of MR was considered for inclusion, both subjective/qualitative measures and objectively measured/quantitative outcomes. Qualitative data were considered for inclusion, given their utility to address complex healthcare questions, such as here around patterns, experiences, and outcomes of MR, thus adding understanding to an area that has been historically understudied.

Any primary randomised or observational study that reported patterns of use and/or outcomes associated with MR was considered. Qualitative studies that employed defined qualitative methodology (i.e. description of recognised approaches to sampling, data collection, and analysis) were eligible for inclusion. Reviews, commentaries of primary studies, and studies that surveyed staff or patient views or perspectives were not considered.

### Data extraction and analysis

A standardised template was used for data extraction by two reviewers (JT and DW), with 20% cross-checked by a third (AL). The level of heterogeneity (e.g. in design, population, outcome, type of MR) was anticipated to be, and found to be, such that quantitative synthesis would not be appropriate, and narrative synthesis was instead undertaken. We predefined a plan whereby when discrepancies between reviewers arose, these would be resolved initially through consensus discussions among the two reviewers, and if necessary, by consulting a third reviewer.

### Quality assessment

For studies reporting the prevalence of MR, the risk of bias was assessed using a checklist developed by Hoy and colleagues [15] and adapted by Agbor and colleagues by removing the criterion for the shortest appropriate prevalence period [16]. For studies focussed on examining the impact of an intervention to reduce the use of MR, the Newcastle-Ottawa scale was used [17].

## Results

### Characteristics of included studies

Searches returned 2,108 unique records, and 309 full texts were reviewed for inclusion (see Supplementary 2 for PRISMA flow diagram). We included 83 articles, which reported on 73 separate studies or datasets. Included studies presented data from between 1990 and 2022, from 22 countries (with some reporting data from multiple countries): 14 from Denmark [14, 18–33], 9 from Germany [34–45], 6 each from Japan [42, 46–50] and Switzerland [44, 51–55], 5 each from China [56–60], Norway [19, 20, 61–65] and Spain [66–71], 4 each from Italy [72–75] and the United States [49, 76–79], 3 from Finland [80–84], 2 each from Australia [49, 85], Belgium [86, 87], Poland [88, 89], Slovenia [90, 91] and The Netherlands [92, 93], and 1 each from Austria [94], Canada [95], Greece [96], Israel [97], New Zealand [49], Nigeria [98], and Scotland [99]. Of 185 data points cross-checked by a second reviewer, there were seven minor discrepancies (96% concordance), all resolved by consensus. Further characteristics are reported in Supplementary 3. See Supplementary 4 for full details of included quantitative studies of rates, associations, and outcomes, and Supplementary 5 for quality assessment of these studies.

### Contemporary studies reporting prevalence of mechanical restraint

**Figure 1.**
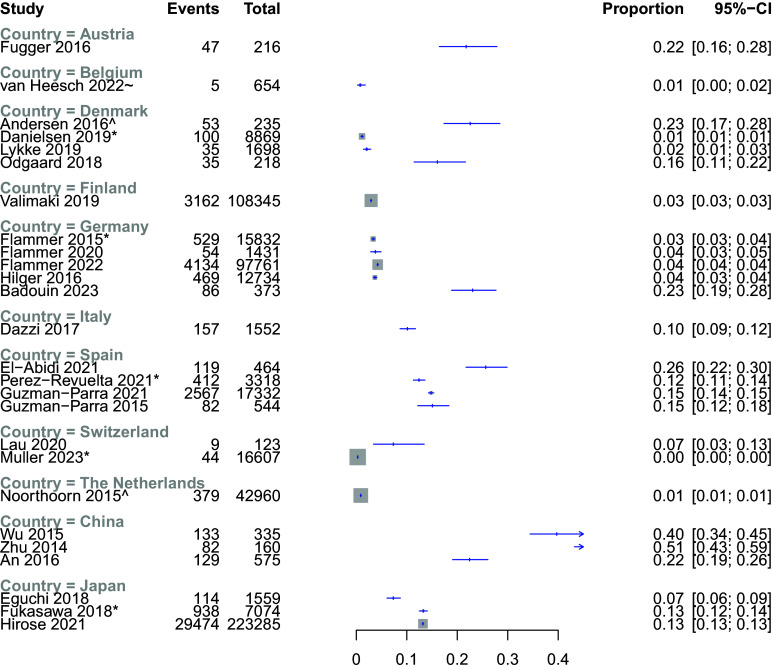
Proportion of patients or admissions (indicated by *) affected be mechanical restraint in included studies (2010 onwards) where this data was reported. ^Mixed adult and forensic samples. ~forensic sample.

Twenty-six studies, conducted in 11 countries, presented prevalence data from 2010 onwards as proportions of all patients or hospital admissions affected by MR (Table 1). We present these for visual comparison in Figure 1, though as per our protocol, we did not pool data. In Europe, prevalence in adult inpatient settings varied between 0.3% in a study in Switzerland [54], up to 26% in one Spanish study [66]. In Japan, individual studies reported a prevalence of 7–13%, whereas the proportion of use was higher in China, ranging from 22–51% in the three included studies. The prevalence of MR also varied within countries.

**Table 1.** Subset of included studies that reported data from 2010-onwards for the proportion of all patients or hospital admissions affected by mechanical restraint (MR). Where studies reported data from a series of years, or pre−/post-intervention, the most recent or post-intervention data was chosen for comparison. SD, standard deviation; IQR, interquartile range.

Study Ref	Last year data collected	Country	Study details, population, setting	Diagnoses	Age	Sex	Restraint device	Information on other restrictive practice	Indications for MR	Total population examined	Prevalence (%) of MR
Fugger, 2016 [94]	2012	Austria	Prospective study of all patients restrained in a psychiatric intensive care unit during study period.	Of 47 restrained patients ICD–10, *n* = 11 for F0, *n* = 6 for F1, *n* = 9 for F20.0, *n* = 4 for F20.2, *n* = 2 for F25.0, *n* = 7 for F31.2, *n* = 1 for F31.6, *n* = 1 for F33.3, *n* = 3 for F50.0, *n* = 3 for F60.3.	Mean 39 (SD 19) of restrained patients.	Mixed, 55% of restrained patients male.	Belt fixation.	Ward has no seclusion rooms.	—	216 patients admitted.	22% (47/216)
van Heesch, 2022 [87]	2020	Belgium	Study of coercive measures in a high security Forensic Psychiatric Center (FPC), including all patients admitted 2014–2020. 83% of patients had a violent index offence, almost all (99%) were in prison prior to admission.	Primary diagnosis psychotic disorder 36%, personality disorder 35%, paraphilic disorder 14%, other 16%.	Mean 42 (SD 12).	Predominantly male (98%).	Any external mechanical devices for limiting movement.	Seclusion in 48%, chemical restraint 12%	In Flemish FPCs, there is a non-MR policy with no restrictive devices being standardly available in wards or seclusion rooms.	654 patients admitted.	1% (5/654)
Andersen, 2016 [18]	2013	Denmark	Two closed psychiatric wards. 18% of patients in study were admitted as forensic psychiatric patients following a hospital order issued by the court.	Schizophrenia primary diagnosis in 56%, affective disorder 10%, substance abuse 9%, personality disorder 8%.	Mean 43 (SD 14).	Mixed, 68% male.	Belt restraint (around waist, securing to hospital bed) +/− strap restraint (wristlets or anklets).	33 (14%) forced medication of whom 20 (61%) also belt- restrained.	May be applied if patient poses a danger to self or others or to inventory in the ward (to a significant degree).	235 patients admitted.	23% belt restraint (53/235). 14% (33/235) also strap restraint.
Danielsen, 2019 [22]	2015	Denmark	Machine learning study to predict MR use in the first 3 days of admission based on analysis of electronic health data, from patients admitted to a psychiatric department from 2011 to 2015.	24% mood disorders, 11% psychotic disorders, 9% substance abuse disorder, 8% anxiety disorder.	35% <30, 25% 30–45, 21% 45–60 (at level of admissions).	Mixed, 51% of admission episodes were of males.	Restraining a patient to a bed using belts or straps.	—	—	5,050 patients with 8,869 admissions.	1% (100/8869) of admissions involved MR 1 –72 hours after admission.
Lykke, 2019 [28]	2012	Denmark	Patients affected by severe mental illness and comorbid substance abuse were hospitalized in 3 large wards (single hospital), 2006–2012.	Substance misuse disorder plus schizophrenia spectrum disorder (50%) or personality disorder (20%).	Mean 40.	70% male.	Fixation by a mechanical device, which includes immobilization with leather belts.	—	Aggression/threatening behavior (41%), extreme agitated state (32%), physical violence toward staff or personnel (15%), destruction of property and endangering self or others (12%).	1,698 hospitalisations.	2% (35/1698)
Odgaard, 2018 [31]	2015	Denmark	Register-based retrospective cohort study of adult inpatients admitted to four wards for affective disorders 2012–2015. Study examined the association between use of the Danish assessment tool for psychiatric inpatients diagnosed with mania (MAS-M) and MR.	The cohort had symptoms of mania/hypomania with or without psychosis (excluded first-time mania) [31].	In those not scored with MAS-M, mean 48 (IQR 34–59), in those scored mean 43 (IQR 31–57).	Mixed, male 45 and 55% in the two groups.	Restraining a patient to a bed by using belt around the waist and/or straps around wrists and ankles to restrict movement.	Only if the patient exposes self/others to immediate bodily harm or danger to health, harasses or molests other patients or commits considerable vandalism.		218 patients admitted.	16% (35/218) restrained in first week of admission, of whom 49% belt only, 51% belt and straps.
Valimaki, 2019 [84]	2014	Finland	Nationwide registry study of adult patients admitted to psychiatric units, examining use of coercive measures 1995–2014. Units offering only forensic psychiatric care were excluded, as were psychogeriatric units.	Any primary psychiatric diagnosis according to ICD–9 or 1CD–10 classifications.	Mean 44 (SD 16).	Mixed, male 52%.	Limb restraint, when a patient may be tied down with belts or comparable tools.	Seclusion 7%, forced injection 3%, physical restraints (holding) 0.8%.	—	In 2010–2014, 108,345 patients admitted.	3% (3162/108345)
Flammer, 2015 [40]	2014	Germany	Aggregated routine electronic data for 7 psychiatric inpatient units.	Main diagnosis as per ICD FO/G3 8%, F1 31%, F2 17%, F3 24%, F4 13%, F5 0.3%, F6 6%, F9 2%.	Mean 46 (SD 19).	Mixed, male 52%.	Use of belts to fix patient to the bed.	Seclusion in 4% admissions, involuntary medication in 78 admissions (0.5%).	—	15,832 admissions of 10,181 patients.	3% of admissions (529/15832).
Flammer, 2020 [37]	2017	Germany	Central register data of 8 forensic hospitals (patients either preliminarily admitted awaiting trial following a crime, or subject to a hospital order).	Main diagnosis as per ICD FO/G3 2.4%, F1 42%, F2 40%, F3 2%, F6 8%, F7 4%, F8 1%.	—	—	Physical restriction of movement by belts.	23% secluded	—	1,431 patients admitted.	4% (54/1431)
Flammer, 2022 [36]	2020	Germany	Study using central register data from 31 licenced adult psychiatric hospitals (excluding forensic).	—	—	—	Freedom-restricting devices: belts in beds, bedrails, movement-restricting blankets, tables attached to a chair.	5% secluded in 2020, 1% forced medication.		97,761 psychiatric hospital cases in 2020.	4% (4134/97761)
Hilger, 2016 [41]	2013	Germany	Retrospective study of an inpatient clinic for patients suffering acute and chronic psychiatric disease, examining restraint and prophylaxis for venous thromboembolism in prolonged restraint (>24 hours).	In prolonged restraint patients, 52% borderline personality disorder, 33% schizophrenia or schizoaffective disorder.	Mean age of prolonged restrained patients 47 (SD 16).	—	5-point fixation – both arms, both legs and trunk.	Did not include those who were secluded (numbers not reported).	—	12,734 patients admitted.	7% (469/12734). 0.3% (36/12734) restrained >24 h.
Badouin, 2023 [45]	2022	Germany	Pre–post study of implementation of peer support in one locked ward compared to treatment as usual in a second locked ward of a psychiatry department.	Schizophrenia (47% intervention, 41% control), substance abuse (27, 39%), affective disorders (7, 9%)	39 (SD 15) in intervention, 39 (12) in control.	Mixed, 62% male in intervention group, 65% male in control.	Fixation via wrist and ankle cuffs attached to the patient’s bed.	8% combined MR and forced medication. 1% forced medication alone.	Situations in which no other means sufficient to prevent further harm, pose a critical threat to patient’s or others’ well-being. Statutory regulations stipulate patient must demonstrate an inability to exercise self-determination.	373 patients in post-intervention analyses.	23% (86/373) 20% (40/200) in intervention group, 27% (46/173) in control group.
Dazzi, 2017 [72]	2013	Italy	Consecutive admissions to an adult Psychiatric Intensive Care Unit.	Schizophrenia 47%, mania 19%, depression 8%, anxiety/adjustment 13%, others 12%	Mean 43 (SD 14).	Mixed, male 48%.	Fixation by belts to a bed.	Seclusion is not used in the ward.	Allowed only in case of actual violent behavior to prevent injuries to the patients or others.	1,552 patients admitted.	10% (157/1552)
El-Abidi, 2021 [66]	2018	Spain	Descriptive study involving a sample of all patients admitted to two acute psychiatry hospitalization units.	Psychotic disorder 69%, depression 12%, substance abuse disorder 5%, others 15%.	Mean 42 (IQR 30–53).	Mixed, male 50%.	Immobilization through devices that cannot be easily controlled or removed.	—	—	464 patients admitted.	26% (119/464)
Perez-Revuelta, 2021 [68]	2014	Spain	Retrospective analysis of MR records on an acute mental health unit 2007–2014, examining risk factors. Also compared with period 2000–2007 to examine impact of organizational measures to minimize use.	Bipolar disorder 15%, personality disorder 15%, psychosis 50%, other 17%.	Mean 42 (SD 13).	Mixed, male 61%.	Wristbands, anklets, belts with magnetic closures and restraint bands to restrict the physical mobility of a patient.	—	Most common indications were agitation (63%) and/or risk of self-harm (58%), or hetero-aggression (65%).	2,448 individual patients admitted 3,318 times.	12% of admissions (412/3318).
Guzman-Parra, 2021 [69]	2018	Spain	Study using MR data from all adult acute psychiatric wards of the Andalusian Health Service from July 2016 to December 2018.	Schizophrenia and psychotic disorders 43%, bipolar disorders 24%, personality disorders 8%, substance use 7%, other 18%.	Mean 42 (SD 14).	Mixed, male 66%.	Application of homologated mechanical fastening devices in beds to limit physical mobility.	No seclusion rooms. No regional registers for pharmacological restraint.	Last resort when all other measures have been ineffective and the safety of the patient, other individuals or the hospital environment is compromised.	17332 people admitted.	15% (2567/17332).
Guzman-Parra, 2015 [71]	2012	Spain	Study of restraint an on acute psychiatric ward in 2005 and 2012, before and after the introduction of a new regulatory protocol designed to reduce the use of restraint.	Psychotic disorders 35%, affective disorders 26%, substance disorders 10%, anxiety disorders 7%, personality disorders 8%, other 7%.	Mean 43 (SD 13).	Mixed, male 59%.	Fastening devices to limit physical mobility.	—	To prevent damage to the patient, other people, and/or the physical environment.	544 people admitted.	15% (82/544).
Lau, 2020 [53]	2018	Switzerland	Longitudinal, observational dynamic cohort study (tracked data in a forensic psychiatric institution, 2010–2018).	90% schizophrenia, of others, 90% substance misuse as secondary diagnosis.	—	Mixed, in 2018 male 87%.	Device used to fixate a patient (e.g. a belt).	In 2018, 19% patients secluded, 9% forcibly medicated.	—	In 2018, 123 patients admitted.	7% (9/123)
Muller, 2023 [54]	2020	Switzerland	Observational study using clinical, procedural, and sociodemographic data from patients treated as inpatients in Switzerland’s largest psychiatric institution 2017–2020.	Substance use disorders 27%, psychotic disorders 24%, depression 21%.	39.9	Mixed, male 56%.	Strapping to a bed with belts with 5-point restraints (arms, legs, and torso) or less.	Other data at level of pooled coercive measures.		8,700 patients with 16,607 admissions.	0.3% (44/16607) of admissions.
Noorthoorn, 2015 [93]	2011	The Netherlands	Observational study using data from hospitals where the Dutch Mental Health Act applies. Included 20 mental health institutes and 3 psychiatric departments of general hospitals covering 75 hospital locations and 375 wards. Covered around 75% of all admissions.	Schizophrenia 32%, drug abuse 26%, personality disorders 26%, mood disorders 23%, organic disorders 3%, neurotic 15%, mental handicap 3%, childhood onset 5%, developmental disorder 5%.	—	—	Use of belts to fix a patient to a bed or chair.	11% seclusion. 0.2% both MR and seclusion, 0.1% MR, seclusion and involuntary medication.	—	42,960 patients admitted.	1% (379/42960)
Wu, 2015 [57]	2014	China (Hong Kong)	Retrospective observational study of patients admitted to the acute psychiatric ward of a public hospital. Recruited with a convenience sample and medical records used to classify into restrained and non-restrained group.	Restraint group: schizophrenia or schizoaffective disorder 27%, paranoid schizophrenia 12%, bipolar disorder 11%, acute psychosis 8%, personality disorder 8%, drug-induced psychosis 9%, depression 8%, mental retardation 9%, dementia 2%, delusional disorder 1%.	Restraint group: 38 (SD 15), non-restraint 44 (SD 17).	Mixed, restraint group 42% male, non-restraint group 44% male.	Safety vests, magnetic limb holders/shoulder straps, pelvic holders, magnetic waist/abdominal belts applied to wrists, ankles, shoulders, waist, and body, or being secured to the bed or chair.	—	—	335 patients admitted.	40% (133/335) restrained in the first 7 days of admission.
Zhu, 2014 [58]	2012	China	Study of all consecutively admitted patients to an adult psychiatric ward who were able to consent.	Schizophrenia 57%, mood disorders 28%, others 15%.	Mean 30 (SD 12).	Mixed, 49% male.	Use of belts to fix a patient to a bed.	—	—	160 patients admitted.	51% (82/160)
An, 2016 [59]	2013	China	Consecutively admitted patients to an adult teaching psychiatric hospital able to give consent, before and after implementation of National Mental Health Law (NMHL).	Schizophrenia 33%, mood disorders 43%, other 24%.	Mean 36 (SD 14).	Mixed, male 36%.	Immobilisation with a mechanical device.	—	If potentially dangerous behaviour was the consequence of a psychiatric disorder, to protect the patient and/or others’ safety, when the patient has refused the necessary treatment in an emergency, e.g. violence or suicide attempt.	575 patients admitted post-NMHL.	22% (129/575)
Eguchi, 2018 [46]	2014	Japan	Retrospective observational study using data from adult patients admitted to emergency or acute wards of a private psychiatric hospital.	All diagnosed with schizophrenia as per ICD–10.	Mean 41 (SD 12).	Mixed, male 44%.	MR using soft belts.	40% seclusion.	Emergency measure to limit behaviour and reactions for managing agitated or violent behaviours.	1,559 patients admitted	7% (114/1559) both secluded and restrained.
Fukasawa, 2018 [47]	2017	Japan	Centralised register data on admissions to general psychiatric wards (excluding forensic) in 113 wards, 23 institutions.	Total sample F0 9%, F1 6%, F2 35%, F3 28%,	—	Mixed, 46% male total sample.	5-point restraints to a bed or a chair on patient’s arms, legs, and torso (fixing a patient at even one point counted).	38% at least one episode of seclusion, excluding older adult.	—	7,074 admissions excluding older adult.	13% (938/7074)
Hirose, 2021 [48]	2017	Japan	Retrospective nested case control study using nationwide registers of patients admitted to psychiatric departments matching patients with and without pulmonary embolism.	In control (no pulmonary embolism), 34% schizophrenia, 33% mood disorder, 6% dementia, 27% other.	In controls median age 51 (interquartile range 31).	Mixed, in controls 39% male.	As per mental health and welfare law in Japan, “restraint with a cloth or band specially made for restraint.”	—	—	223,285 patients, 660 case–control pairs matched by age and sex from same facility in same year were generated.	13% (29474/223285)

### Studies of forensic populations

Among the 10 studies that explicitly included forensic patients, one German study included 1,431 patients admitted across eight forensic hospitals, examining restraint compared with general psychiatric wards [37]. MR with belts affected 4% of patients in forensic wards, slightly lower than in the general psychiatric wards. However, the proportion of patients subject to seclusion (23%) was around 8-fold higher in the forensic wards than in general psychiatric hospitals. A Dutch study in which the overall use of restraint was very low (<1%) reported that restraints were primarily on forensic rather than general wards [93]. Similarly low rates of MR were reported in a study of a high-security forensic setting in Belgium, where out of 654 patients admitted over six years, five (0.8%) were mechanically restrained [87]. This is in the context of a clear local policy for no MR- in contrast, 48% of included patients were secluded. Two studies of forensic settings used qualitative methods to examine patient and staff perspectives [33, 99], or examined the impact of interventions to reduce restraint in forensic settings [30, 78, 79], discussed below.

### Quantitative studies of rates, associations, and outcomes

#### Patterns and indications

Indications for MR were typically broad across included studies, principally for physical violence, threats, or aggression, or for significant danger to self or property. There was limited comparison of outcomes when restraint was used for different indications, although a study of 371 restrained patients in Norway reported those who were mechanically restrained for self-injury were restrained for significantly shorter periods than for other reasons [62, 63].

In some cases, local policy dictated that physical violence was the only indication for use [72]. Local policy emphasis appeared to be related to the prevalence of use. For example, in one Swiss study, ward policy stated it was for “highly exceptional” use, with a preference instead for seclusion and forced medication. MR in this setting was low (0.5% of admissions) [51]. In contrast, in one Italian psychiatric intensive care setting, seclusion was not available, and here 10% of patients were restrained at least once [72]. A smaller number of studies also referred to specific additional indications for MR, such as to permit treatment [97] or to reduce absconding risk [84], including in a planned manner for offsite transfers.

Studies reporting patterns in the use of MR considered numerous factors. Most consistently, in acute adult psychiatric settings, the early phase of admission (hours and days) was the period of highest risk for restraint [18, 68, 73, 74]. In many cases, significant variation was found in use between different periods of the day and night, but the pattern of this varied between studies. Some reported less frequent use during the morning and afternoon shifts compared with the night shift [73]. Other studies found restraint occurring in other patterns, such as more often at night [74], with morning and evening peaks [97], similarly distributed across day and night shifts [72], or in the evening shift [24]. This included one Danish study (using data from 5,456 episodes of MR) in which restraint was initiated more often in evening than in day shifts (and with fewer episodes initiated at night for all types of coercion) [25-27]. Another Danish study found that restraint was predominantly implemented during the day (8 am‐4 pm) and evening (4 pm‐12 am) shifts (82%), and only administered 18% of the time in the early morning when staff-patient ratios were lowest [28].

One Norwegian study included 19,283 patients admitted to acute psychiatric settings over eight years and found that the use and type of restraint varied significantly by seasonal time [65]. During summer, MR was used significantly more often than pharmacological restraint. A Danish study also found a significant variation by month of the year [24].

#### Clinical and demographic factors

Among the more consistent findings was an association of restraint and duration of restraint with male sex [50, 63, 64, 72, 95] and younger age [72, 95]. Risk factors for restraint in individual studies also typically aligned with clinical factors associated with increased violence risk, such as persecutory ideation [22], intoxication [18], poorer insight [59], and Broset violence checklist score [22].

The relevance of ethnicity or immigrant background was examined by several studies. A Norwegian study reported patients from ethnic groups other than Norwegian had a lower risk of restraint (odds ratio [OR] 0.4, 95% CI 0.2–1.0) [61] and an inverse association with ethnicity was also reported by a study including 42,960 patients in The Netherlands [93]. A Spanish study of 474 people consecutively admitted to acute wards found that language barrier was associated with a higher risk of MR (OR 2.1, 95% CI 1.2–3.7) [66]. An Italian study reported that extra-European nationality was associated with restraint [74], and another study in Italy examined this relationship directly by matching 100 first-generation immigrants with 100 non-immigrants, finding that immigrant patients were more likely to be restrained as compared to Italian-born patients (11% vs 3%, relative risk [RR] 3.7, 95% CI 1.1–12.7) [75]. No significant differences were found between groups in rates of repeated restraints, however, nor in the overall duration of restraint, a finding mirrored by a study in Norway [62, 63].

Several protective factors were reported, such as prior community mental health contact [18], negative symptoms, and negative affect [72]. In a study comparing a total of 2,927 episodes of restraint in Denmark and Norway, mandatory review, patient involvement, and lack of over-crowding were significantly associated with a low frequency of MR episodes, and six preventive factors confounded the differences found between the countries: staff education, substitute staff, acceptable work environment, separation of acutely disturbed patients, patient:staff ratio, and the identification of crisis triggers [19, 20].

#### Staff factors

Few studies reported on associations with staff or ward factors. A study in Japan of 7,074 admissions found restraint (and seclusion) was more likely in wards with more beds, more nurses, in acute wards, and in urban areas [47]. A Danish study of 259 admissions found an association with the male gender of care workers (OR 1.4, 95% CI 1.0–2.1) but no associations were found between restraint and staffing level, age, education, experience of care workers, or change of shifts [24].

#### Outcomes and acceptability

One randomized trial compared experiences of coercion with MR versus seclusion in an adult admission ward [34]. Patients were interviewed four weeks after the intervention and re-interviewed around 18 months later in a follow-up study [35]. Factors most frequently cited by patients to alleviate the distress associated with restraint were contact with staff and having personal objects nearby. In the original study, there were no significant differences in experience of stress between the two groups, in adverse events, or in the level of experienced coercion. At follow-up, however, coercion ratings for MR versus seclusion were significantly more negative on six of the nine items.

A Danish national study examined all complaints received via their centralised system. Roughly one in six patients subject to MR filed a complaint, and for around one in 25 restrained patients, this was subsequently found to have been illegitimate when reviewed by authorities (typically as no violence or threat was demonstrated) [21]. Several studies quantitatively assessed patients’ experiences of coercion or trauma related to restraint. An Austrian study interviewed patients shortly after restraint. On visual analogue scales, patients considered themselves depressed and powerless during restraint, with fear relatively absent. Anger was markedly present during restraint but not in consecutive visits as psychopathology improved. Patients’ acceptance of the coercive measure was higher than expected, while patients’ memory was significantly lower. About 50% of the patients documented high perceived coercion, and PTSD could be supposed in a quarter of the restrained individuals [94]. Another Danish study assessed 20 patients who had each experienced multiple MR episodes, and in this sample, interpretation of restraint episodes as central to identity was significantly related to higher PTSD symptoms [23]. Centrality of episodes also explained variation in PTSD symptom severity. A study in Spain of 111 people who had been restrained and/or involuntarily medicated found significant differences in experienced coercion, this being highest in combined measures followed by those who had been mechanically restrained [67].

Two studies examined rates of venous thromboembolism. In a German study in which 469 patients were restrained, none of the restraints (either prolonged, in which case patients are given prophylaxis with enoxaparin, or those lasting less than 24 hours, who are not given prophylaxis) were associated with deep vein thrombosis [41]. However, a Japanese study including 660 case–control pairs of patients found that being in physical restraint for 15+ days was associated with increased risk of pulmonary embolism (OR 3.2, 95% CI 1.2–8.5) [48].

There was very limited reporting of measurable positive effects. Japanese data in patients with psychosis where seclusion with restraint was used reported favourable changes in psychosis and thought disorder as measured by the Brief Psychiatric Rating Scale (BPRS) [46].

### Impact of interventions, policy, or other changes

Among the 16 studies reporting the effects of changes (Supplementary 6, and Supplementary 7 for quality assessment), no significant effect was reported for moving to a new hospital building [29], use of an assessment tool for psychiatric inpatients diagnosed with mania [31], sensory modulation [32], or peer support [45]. A study of implementing moral case deliberation (reflective practice) on two wards in Switzerland showed no significant decrease in the number of restraints, though the intensity of restraints (calculated using the duration) did significantly decrease [55]. A Danish study of the implementation of the Safewards model showed no effect, but trends were already following a downward trajectory prior to the study period [14], and another Polish study of Safewards did show a significant difference in the number of patients mechanically restrained [89].

Other studies showed the impact of legislative or policy changes. A Chinese study examining restraint before and after the implementation of a national mental health law found that restraint was independently associated with having been admitted before the law change [59]. In a German study, the introduction of the requirement for an immediate judge’s decision for any restraints lasting longer than 30 minutes was associated with a significant reduction in restraint (but an increase in seclusion) [38].

In eight Danish forensic units, a stepped-wedge cluster-randomised trial examined the implementation of the short-term assessment of risk and treatability (START) to reduce MR in male patients who displayed at least one aggressive episode [30]. This was associated with a significant reduction in MR (RR 0.2, 95% CI 0.1–0.4). A cluster randomised trial of the implementation of de-escalation training in Slovenia was also associated with a reduction to 30% of the rate in the control group (incidence rate ratio [IRR] 0.3, 95% CI 0.2-0.4) [91].

Other studies examined the impact of more cumulative changes. A large Spanish study including data from over 17,000 people admitted described changes associated with a multicomponent intervention based on the “Six Core Strategies.” [69] Comparing the first and last semester of the study, there was a significant reduction in restraint hours (by 33%), restraint episodes (by 6%), and proportion of patients restrained (by 8%). There was a significant decreasing trend in the total number of MR hours during the implementation of the intervention, but not in the number of episodes [69].

Similarly, an American study described the impact over two 10-year periods of multiple measures, demonstrating a significant decline in the use of restraint in forensic centres in Pennsylvania [78, 79]. During the decade to 2010, the rate of patient-to-staff assaults declined, though the rate of patient-to-patient assaults was unaffected. Leadership, data transparency, use of clinical alerts, workforce development, policy changes, and discontinuation of psychiatric use of as-required medication orders were all described as contributing factors [78]. In the subsequent decade, seclusion and restraint were abolished entirely, and incidents of assault, aggression, and self-injurious behaviour significantly declined or were unchanged by the decreasing use of containment procedures [79].

#### Qualitative studies

Findings from four included qualitative studies [33, 60, 98, 99] are detailed in Supplement 8.

## Discussion

This review represents the most extensive synthesis to date of published studies examining the use of mechanical restraint (MR) in inpatient psychiatric settings internationally. It addresses evidence gaps in previous work by using more exhaustive search criteria focussed on MR, and considering a full range of adult inpatient settings. In so doing, we have presented data from 73 different studies of mechanical restraint, substantially expanding on existing syntheses [4, 7], which have either undertaken broader examinations of restrictive practice or focussed on the small number of comparative studies. We present four key summary findings from this new, comprehensive review with implications for clinical services, policymakers, and researchers.

First, for the first time assimilating prevalence data in this manner, we have demonstrated the extent to which MR in adult inpatient psychiatric wards internationally remains widely practiced. Individual studies reporting the prevalence of use since 2010 provide estimates ranging to an upper bound of 13% in Japan, 27% in a European setting, and 51% in China. This intervention thus requires regulation and a clear consensus on best practice to support frontline staff, who must consider complex ethical issues to balance autonomy, dignity, and safety [100]. This guidance should be based on a robust appraisal of outcomes alongside human rights considerations. Prevalence varied widely between included studies, including between hospitals within the same countries and regions. Differences are therefore likely attributable in many cases to hospital-level policy variation.

Second, MR was broadly defined in most included studies as the use of belts or straps, with limited granularity in the description (e.g. manufacturer, exact materials), indications for use, and outcomes associated with different types of MR. Importantly, despite the widespread use, many included studies did not give a clear account of the specific indication for MR, compared with other forms of restrictive practice. Where this information was available, local policy, rather than clinical or other factors, appeared to guide practice. For instance, where one or other form of restriction was either preferred or was unavailable (such as in centres/regions in which seclusion rooms were not present), this appeared to largely account for any very low rates of use of one or other form of restriction in included studies. Local policy and legislation around approval and review may also account for the apparent variations in the length of time spent in restraint.

Third, studies provided limited insight into the influence of clinical and demographic factors. Factors such as younger age, male sex, and substance misuse were the most consistently associated with MR. This is understandable theoretically, given the overlap with established violence risk factors in psychiatric populations [101, 102], and the finding that violence was typically defined as one of the main indications for MR in included studies. In acute settings, the early phase of admission was identified as a higher risk for MR. However, other potentially modifiable factors associated with the use of MR were examined to only a very limited extent, such as the impact of staff factors and shift patterns, which was reported in several studies, but without clear consensus. Such factors are likely to be highly unit-specific and are important to understand given they may lend themselves to being practically addressed. Language barriers and ethnicity or immigrant status were also identified as potentially important avenues for further exploration. The positive impact of strategies to develop staff skills in verbal de-escalation would seem to triangulate with the importance of communication in avoiding the need for MR.

Fourth, data regarding outcomes associated with MR was limited, while studies that compared MR directly with other forms of restriction in terms of outcomes were rare. Only one randomized study directly compared restraint with seclusion, and whilst post-intervention assessment of affected patients did not find a significant difference between groups, follow-up after 18 months found the restraint to be significantly less favorably regarded than seclusion. Findings from other studies of perceived coercion and PTSD symptoms also identified these as areas for consideration. Regarding physical sequelae of restraint, prolonged restraint was associated with pulmonary embolism risk but there was limited other reporting of physical health outcomes.

### Implications for clinical practice and future research

Included studies highlight key areas that require further examination in both reviews of local clinical practice and future empirical research.

Detailed case-use mapping of the type, duration, and specific indications for restraint in different settings and diagnostic profiles should be a priority. Whilst risk to others broadly is the most frequently cited indication, a consensus around the typical scenario for which MR is of benefit over other forms of coercion is not well described, other than *in extremis*, in settings where other forms of coercion are preferred as the first line. References to the principles of collaborative risk assessment and management, which are increasingly seen as policy priorities, were notably lacking in included studies For example, instances where MR has been pre-planned or part of an agreed individual care-plan were not described in the included studies. Similarly, approaches to monitoring physical wellbeing whilst in restraint were not well described in the included studies and these need development and practical evaluation.

There was a suggestion in included studies that language, communication barriers, and ethnicity warrant exploration as potential risk factors. Such factors are likely to vary in their significance in local contexts, and so should be a focus for local clinical services as well as larger-scale research. Likewise, the relation of ward staff mix (gender, ratios, shift changes, and times of day) needs examination given evidence for their potential relevance to patterns.

High-quality studies of patient experience were limited and this should be a priority for future research [103, 104]. Such work would benefit from being assessed as proximally to the restraint incident as possible to avoid recall bias, and the small number of included studies that used this approach demonstrated that this is feasible. Included studies did provide examples of best practice or factors that either reduced the need for or improved the experience of restraint that require further clarification and standardised implementation. These included processes for mandatory review or patient involvement, interaction style of staff, and frequency of contact during restraint, along with explanation and the presence of personal belongings. More broadly, staff permanency, ratios, and satisfaction were associated with lower levels of restraint and are of importance at a service level.

Positive outcomes (such as improvement in psychotic symptoms) were seldom reported in the included studies. Understanding these, as well as the reduction of negative outcomes such as assault, for an individual patient, compared with other forms of coercion, requires individualised consideration. Only one study examined staff experiences [99], and for an intervention that requires such direct physical involvement by staff, this is a significant knowledge gap that needs addressing.

Several studies reported changes that significantly reduced or even abolished MR. In keeping with the wider literature for reducing restrictive practice [105], the nature of these interventions in included studies was heterogeneous, and evidence mixed. There was, however promising evidence for implementation of ward-level interventions such as de-escalation training or assessment tools where this was with the specific goal of reducing MR. Specifically targeted procedural changes, such as to the legal approval framework for ongoing restraint, also had a significant effect. Overall, there was an indication that rates of MR are sensitive to change in individual units. Such work however cannot be interpreted without understanding aligned changes in other forms of coercion. Further research is also needed to understand whether reductions are specifically attributable to the intervention or a general effect of increased scrutiny during such periods.

## Conclusion

Mechanical restraint remains widely practiced in psychiatric settings internationally, though with considerable national and regional variation. Given the clinical and ethical implications, robust empirical support for its use is essential, and clinical policy should be evidence-led rather than based only on local conventions or facilities. High-quality studies remain scarce, especially those specifying the type of restraint, indications, clinical factors associated with use, and impact of ethnicity and language (of both patients and staff). Evidence for outcomes is even more limited, with little or no high-quality evidence of patient experience. These considerations should be research priorities, as such work has the potential to directly influence improved best practice guidelines. In limiting the use of mechanical restraint, some ward-level interventions show promise, however, strategies must be considered in the context of other restrictive practices, including seclusion. While abolishing mechanical restraint in psychiatry may not be realistic, there is evidence to suggest it is possible to improve the precision, safety, and effectiveness of its use. This should encourage further high-quality studies, which are imperative in aligning this practice with expected clinical and ethical standards of contemporary psychiatric care.

## Supporting information

Whiting et al. supplementary material Whiting et al. supplementary material

## Data Availability

Data from included primary studies supporting the findings of this review are contained within the manuscript and supplementary material.

## Supplementary material

The supplementary material for this article can be found at http://doi.org/10.1192/j.eurpsy.2025.2453.
