# Supplementary material for: Mechanical restraint in inpatient psychiatric settings: A systematic review of international prevalence, associations, outcomes, and reduction strategies
Source: Eur Psychiatry. 2025 Apr 25;68(1):e57. doi: 10.1192/j.eurpsy.2025.2453 (PMC12090036; doi:10.1192/j.eurpsy.2025.2453)
Supplement: Whiting et al. supplementary material [file S0924933825024538sup001.pdf]

Mechanical Restraint in inpatient psychiatric settings: A systematic review of international prevalence, associations, outcomes and reduction strategies

Daniel Whiting, Alexandra Lewis, Kursoom Khan, Eddie Alder, Gill Gookey and John Tully

Supplementary Materials

Supplement 1

Search strategy.

**Platform and database**: Ovid Medline® ALL <1946 to September 07, 2023>		
**Link to run search:** https://access.ovid.com/custom/redirector/wayfless.html?idp=https://idp.eng.nhs.uk/openathens&url=http://ovidsp.ovid.com/ovidweb.cgi?T=JS&NEWS=N&PAGE=main&SHAREDSEARCHID=11RqlIbQ8EPOZgHg5WB2pUWWarj7dhi6Hq1DbT0QxjeLZxa31ZUODnV4jBLxVIdyj		
1	*Restraint, Physical/	4521
2	(mechanical* adj3 (restrain* or device* or fastening* or intervention*)).ti,ab.	8033
3	(restrictive adj (intervention* or practice*)).ti,ab.	331
4	((physical* or forced) adj (restrain* or immobili*)).ti,ab.	2671
5	((device* or belt*1 or cuff*1 or strap*4 or jacket* or vest* or mitt*1 or mitten* or holder* or chair* or anklet* or rail*1) adj4 (restrain* or restrict* or subdu* or immobili*)).ti,ab.	3330
6	1 or 2 or 3 or 4 or 5	17341
7	Forensic Psychiatry/	9433
8	Hospitals, Psychiatric/	26081
9	Psychiatric Nursing/	18243
10	Mental Disorders/	178166
11	((high or medium or low or regional or maximum) adj secur*).ti,ab.	1883
12	((forensic* or secure or mental or psych*) adj3 (hospital* or facilit* or unit* or service* or setting* or ward* or institut* or healthcare or "health care" or patient* or inpatient* or in patient* or profession* or staff* or nurs* or doctor* or worker*)).ti,ab.	214441
13	forensic psychiatr*.ti,ab.	3573
14	((mental* or psychiatric*) adj2 (disorder* or illness* or condition* or disease* or diagnos*)).ti,ab.	179986
15	7 or 8 or 9 or 10 or 11 or 12 or 13 or 14	466608
16	6 and 15	1509
17	limit 16 to english language	1332

**Platform and database**: Ovid Embase <1974 to 2023 September 07>		
**Link to run search:** https://access.ovid.com/custom/redirector/wayfless.html?idp=https://idp.eng.nhs.uk/openathens&url=http://ovidsp.ovid.com/ovidweb.cgi?T=JS&NEWS=N&PAGE=main&SHAREDSEARCHID=5gvrgmjxLWiqhMDBkcij99oMGcUEqeriqyN4LmAOERTGECv3VHaFKlFwXoScFo9DN		
1	*physical restraint/	312
2	(mechanical* adj3 (restrain* or device* or fastening* or intervention*)).ti,ab.	11291
3	(restrictive adj (intervention* or practice*)).ti,ab.	423
4	((physical* or forced) adj (restrain* or immobili*)).ti,ab.	3396
5	((device* or belt*1 or cuff*1 or strap*4 or jacket* or vest* or mitt*1 or mitten* or holder* or chair* or anklet* or rail*1) adj4 (restrain* or restrict* or subdu* or immobili*)).ti,ab.	4387
6	1 or 2 or 3 or 4 or 5	19228
7	forensic psychiatry/	13533
8	mental hospital/	26218
9	psychiatric nursing/	15758
10	mental disease/	277395
11	((high or medium or low or regional or maximum) adj secur*).ti,ab.	2718
12	((forensic* or secure or mental or psych*) adj3 (hospital* or facilit* or unit* or service* or setting* or ward* or institut* or healthcare or "health care" or patient* or inpatient* or in patient* or profession* or staff* or nurs* or doctor* or worker*)).ti,ab.	289113
13	forensic psychiatr*.ti,ab.	4871
14	((mental* or psychiatric*) adj2 (disorder* or illness* or condition* or disease* or diagnos*)).ti,ab.	243657
15	7 or 8 or 9 or 10 or 11 or 12 or 13 or 14	635851
16	6 and 15	1310
17	limit 16 to english language	1196
**Platform and database**: ProQuest APA PsycInfo®		
S1	MJMAINSUBJECT.EXACT("Physical Restraint")	1816
S2	ti,ab((mechanical or mechanically) NEAR/3 (restrain* or device* or fastening* or intervention*))	708

S3	ti,ab(restrictive NEAR/1 (intervention or interventions or practice or practices))	406
S4	ti,ab((physical or physically or forced) NEAR/1 (restrain* or immobili*))	1436
S5	ti,ab((device* or belt*1 or cuff*1 or strap*4 or jacket* or vest* or mitt*1 or mitten* or holder* or chair* or anklet* or rail*1) near/4 (restrain* or restrict* or subdu* or immobili*))	316
S6	[S1] or [S2] or [S3] or [S4] or [S5]	3783
S7	MAINSUBJECT.EXACT("Forensic Psychiatry")	8469
S8	MAINSUBJECT.EXACT.EXPLODE("Psychiatric Hospitals")	10723
S9	MAINSUBJECT.EXACT("Psychiatric Nurses")	4005
S10	MAINSUBJECT.EXACT("Mental Disorders")	145443
S11	ti,ab((high or medium or low or regional or maximum) NEAR/1 (secure or security))	3349
S12	ti,ab((forensic or forensics or secure or mental or psych or psychiatric or psychiatry) NEAR/3 (hospital or hospitals or facility or facilities or unit or units or service or services or setting or settings or ward or wards or institute or institution or institutions or healthcare or "health care" or patient or patients or inpatient or inpatients or "in patient" or "in patients" or profession or professions or professional or professionals or staff or nurse or nurses or nursing or doctor or doctors or worker or workers))	156841
S13	ti,ab("forensic psychiatry" or "forensic psychiatrist" or "forensic psychiatrists")	2498
S14	ti,ab((mental or psychiatric) NEAR/2 (disorder or disorders or illness or illnesses or condition or conditions or disease or diseases or diagnosis or diagnoses or diagnosed))	161955
S15	[S7] or [S8] or [S9] or [S10] or [S11] or [S12] or [S13] or [S14]	345423
S16	[S6] and [S15]	1111
S17	([S6] and [S15]) AND la.exact("ENG")	1025

Supplement 2

PRISMA flow chart.

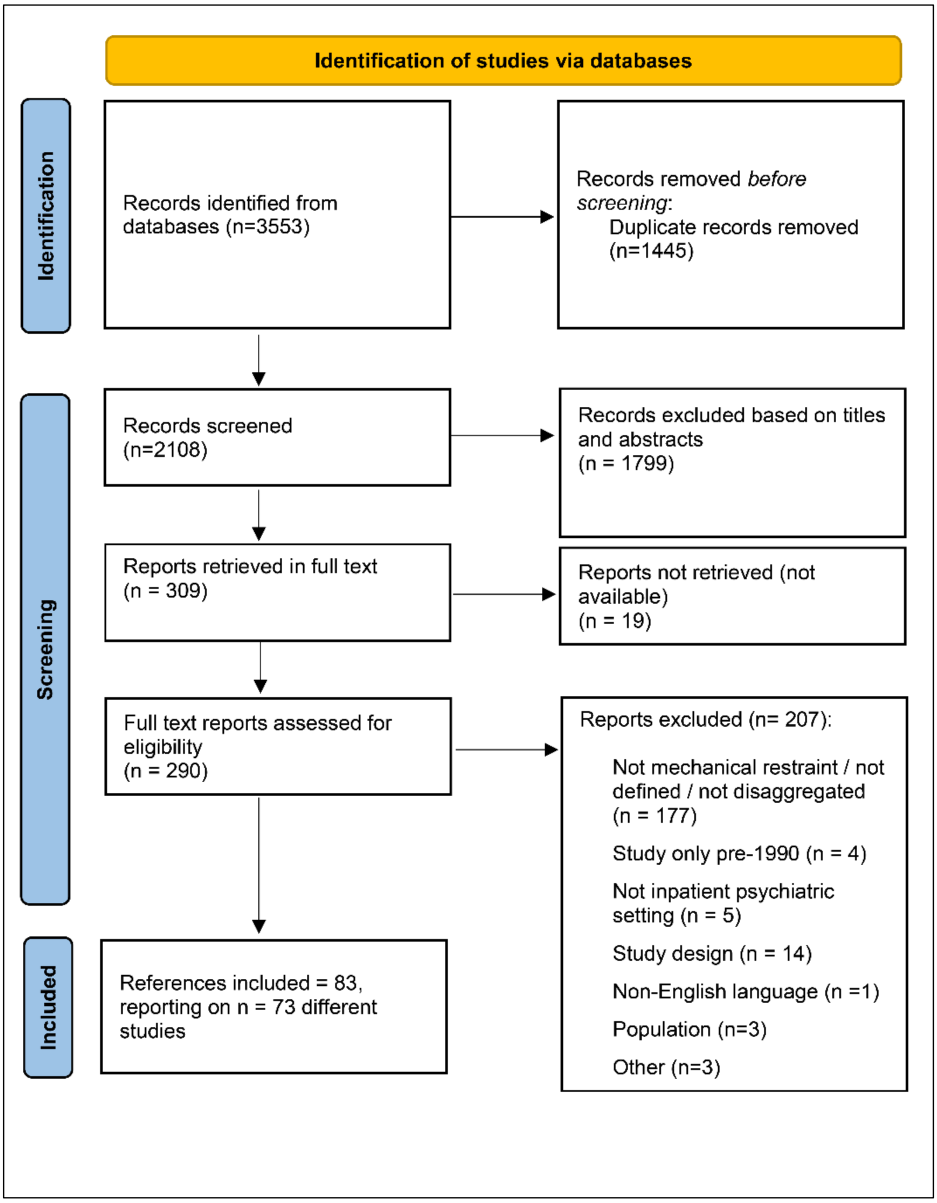

Supplement 3

Further characteristics of studies

Of the 73 separate included studies, 53 focussed on outcomes, rates or associations with mechanical restraint (MR) (Supplement 4). Of the 46 studies in this group that reported overall quantitative rates of MR, 5 (11%) were assessed as being at moderate risk of bias, and 45 (89%) as at low risk of bias (Supplement 5). Sixteen studies examined changes following interventions targeting reducing coercion and other system-level or legislative changes, or described sequelae from ceasing use of MR (Supplement 6). Of these, 10 (63%) were rated as good quality, and 6 (38%) as poor quality (Supplement 7). Four studies used qualitative methods to examine staff or patient experience of defined episodes of MR (Supplement 8). Overall, most studies (63/73, 86%) were from general adult inpatient psychiatric settings. Ten studies (reported in 11 publications) specifically included forensic populations, either as the sole population,(1-8) or as part of the overall sample.(9-11)

Typically, studies included defined MR broadly as a movement-restricting device, usually leather straps or belts to restrict movement via the limbs or waist, with or without restraining to a bed or chair. In a small number of studies, other specific measures were included within definitions, including the use of gloves,(12-15) soft belts including Velcro,(4, 5, 16) handcuffs,(17) vests,(17-19) bed rails,(20, 21) movement-restricting blankets,(20-22) special cloths or bands,(23) straitjackets,(24-26) camisoles or restraining sheets,(27) net restraints,(22) pelvic holders,(18) magnetic shoulder straps,(18, 28) and chains.(29)

Supplement 4

Quantitative studies of rates, associations and outcomes of mechanical restraint (MR). Empty cells, marked with (-), indicate that information was not reported.

Study Ref	Last year data collected	Country	Study details, population, setting	Diagnoses	Age	Sex	Restraint device	Prevalence of other forms of coercion	Total N of population examined	Prevalence (%) or rate of MR	Indications for MR	Patterns, associations and outcomes of MR
Andersen, 2016	2013	Denmark	Two closed psychiatric wards. 18% of patients were admitted as forensic psychiatric patients following a hospital order issued by the court.	Schizophrenia primary diagnosis in 56% total sample, affective disorder 10%, substance abuse 9%, personality disorder 8%.	43 (SD 14).	Mixed, 68% male.	Belt restraint (around waist, securing to hospital bed) +/- strap restraint (wristlets or anklets).	33 (14%) forced medication of whom 20 (61%) also belt-restrained.	235	Belt restraint in 23%, 33 (14% of total) also strap restrained.	May be applied if the patient poses a danger to self or others or to inventory in the ward (to a significant degree), even if the patient is not psychotic.	Of 53 patients belt-restrained, 78% were restrained during in first 24 h and 42% in first hour following admission, especially if admitted on basis of danger criterion (20 (62%) restrained within the first hour).<br><br>Belt restraint significantly positively associated with involuntary admission, acute intoxication, and negative association with community mental health contact.
Bak, 2014<br><br>&<br><br>Bak, 2015	2010	Denmark & Norway	All psychiatric hospital units in Denmark (87) and Norway (96) that treated adult inpatients (18–65 years) in which MR had been used for approximately two years prior to the year of investigation were included.<br><br>Examined ward-level factors that were associated with lower rates of MR, and with the difference between countries.	-	18-65.	-	Restraining straps, belts, or other equipment to restrict movement	-	1,938 restraint episodes in Denmark and 989 in Norway were analysed.	Restraint episodes during 1 year per unit varied between 0 and 254, mean 32.5. In Denmark mean was 45.1, in Norway 21.0.	When necessary to prevent damaging/ exposing their or others' body/health to danger, in any other way grossly molesting fellow patients, or committing significant acts of vandalism.	Mandatory review (exp(B) = .36, p < .01), patient involvement (exp(B) =.42, p < .01), and no crowding (exp(B) = .54, p < .01) were significantly associated with a low frequency of MR episodes.<br><br>Six preventive factors confounded the difference between Denmark and Norway: staff education, substitute staff, acceptable work environment, separation of acutely disturbed patients, patient – staff ratio, identification of the patient's crisis triggers.
Bergk, 2011<br><br>&<br><br>Steinert, 2013	2006	Germany	Comprehensive cohort study of two randomised and two non-randomised cohort arms, to compare experiences of coercion with MR versus seclusion, in inpatients aged over 18 in a psychiatric admission ward able to consent and recall index intervention.<br><br>Follow-up interviews of patients around 18 months after original randomised study.	Schizophrenia, affective disorder or personality disorder. In follow-up study, schizophrenic disorder 63%, bipolar disorder 23%.	Mean 39-41 across groups with no significant differences. In follow-up study, mean 40 (SD 13).	Mixed, 49% male. In follow-up, mixed, 55% male.	Five-point restraints in a bed (both arms, both legs, and a hip belt).	-	102<br><br>26 randomly assigned to seclusion (N=12) or MR (N=14), 76 excluded from randomization and included in the cohort arms (28 experienced MR).<br><br>60 (59%) from original sample were available for re-interview.	-	In follow-up study, most coercive measures were carried out because of threatening behaviour (N=39, 65%), followed by danger to self and danger to property, with no significant differences between secluded and restrained patients.	In follow-up study, when asked what alleviated distress, patients most frequently mentioned contact with staff (N=20, 68%) and having personal objects nearby (N=3, 11%).<br><br>No significant difference between groups in the level of experienced coercion as measured by the Coercion Experience Scale (CES). Patients in the seclusion group received less medication than those in the restraint group.<br><br>During MR episodes, 1 adverse event was observed: retraumatization of a former torture victim. No significant differences between the seclusion and MR groups in adverse events.<br><br>In follow-up study 12 (41%) who experienced MR retrospectively judged the measure as justified. Others complained about inhuman practice, arbitrariness, or disproportionality.<br><br>Ratings for MR had changed significantly in follow-up study for violation of human dignity, restriction of freedom to move, experience of restriction of freedom to move, autonomy, coercion at beginning of measure, and experience of coercion (CES ratings became more negative).
Bilanakis, 2010	2007	Greece	Chart review of consecutive admissions to a general adult psychiatric inpatient unit.	Schizophrenia 48%, affective disorder 26%, personality disorder 9%, substance abuse 9%.	92% aged 19-60.	Mixed, 55% male.	Use of belts to secure a patient to a bed.	-	282 admissions	1.8% mechanically restrained	Threat to self or to others and severe disrupted behaviour	-
Birkeland, 2018	2014	Denmark	Audit of all complaints received by the national complaint board regarding MR 2007-2014.	-	-	-	Belt, hand/foot straps, and gloves	-	163 cases where complaints filed regarding restraint.	-	56% violence or threats, 17% physical violence toward staff/patients, 13% self-destructive, 25% verbal threats, 28% other violent (e.g. psychological) or aggressive behavior.	During the study period, roughly every sixth patient subject to MR filed a complaint and for 1 in 25 patients subject to restraint this was found illegitimate by authorities (in 89% as no violence/threat demonstrated). Duration found illegal in 33 cases (20%). Documentation explicitly found insufficient in 39 cases (24%).

Chieze, 2021	2017	Switzerland	Retrospective study of every patient hospitalised in adult psychiatry division of 6 inpatient wards for adults.	Psychotic disorder 57%, bipolar disorder 19%.	18-65	Mixed, 53% male.	Five-point belts, immobilization, waist-belts, ankles and wrists fasteners, bed-rails.	228 of hospital stays (16%) involved seclusion, and 16 (1%) involved forced medication.	865 patients, with 1405 hospital stays.	7 hospital stays (0.5%) involved restraint.	When coercion needed, division policy recommends seclusion and forced medication rather than restraint, which is reserved for highly exceptional situations.	-
Danielsen, 2019	2015	Denmark	Machine learning study to predict MR use in the first 3 days of admission based on analysis of electronic health data after the first hour of admission, from patients admitted to a psychiatric department 2011-2015.	24% mood disorders, 11% psychotic disorders, 9% substance abuse disorder, 8% anxiety disorder.	35% <30, 25% 30-45, 21% 45-60 (at level of admissions).	Mixed, 51% of admission episodes were of males.	Restraining a patient to a bed using belts or straps.	-	5050 patients with 8869 admissions.	100 admissions (1%) involved MR between 1 hour and 72 hours after admission.	-	Most important predictors: involuntary admission, Broset violence checklist score, somatic comorbidity, non-coherent verbal responses, abnormal or threatening behaviour, good social status, suicidal ideation, persecutory ideation.
Dazzi, 2017	2013	Italy	Consecutive admissions to an adult Psychiatric Intensive Care Unit.	Schizophrenia 47%, mania 19%, depression 8%, anxiety/adjustment 13%, others 12%.	Mean 43 (SD 14).	Mixed, male 48%.	Fixation by belts to a bed.	Seclusion is not used in the ward.	1552	157 (10%) restrained. Of these, 29 patients (19%) needed to be repeatedly restrained.	Allowed only in case of actual violent behavior to prevent injuries to patients or others.	Similar distribution across day and night shifts (118 episodes during the day shift, 104 at night).<br><br>In multivariate analysis, independent predictors of restraint: male, younger age, compulsory admission, CGI score, resistance/activation/disorganization on BPRS. Negative symptoms and negative affect were protective.
De Hert, 2010	2009	Belgium	Consecutive admissions to 11 inpatient adult wards at a psychiatric hospital.	All had schizophrenia.	Mean 42 (SD 14).	Mixed, male 69%.	One- to five-point restraint, confining limbs on a specially designed bed.	170 (25%) were secluded	679 patients (1310 admissions)	138 (20%) at least one restraint (all were whilst secluded).	-	-
Di Lorenzo, 2012	2008	Italy	All patients subject to restraint admitted to a public psychiatric ward at a university hospital affected by acute psychiatric diseases, between 2005-2008.	Schizophrenia and psychotic disorders, alcohol/substance-related, mood disorders, personality disorders, adjustment reaction, mental retardation, dementia (proportions not reported at total population level).	Median age of restrained patients 36 in 2008.	Mixed, male 40-60% of restrained patients over the years of study.	Mechanical containment by handcuffs, leg ties, and vest to immobilize the patient in bed.	In 50% of the restraints a supplementary therapy was prescribed: sedative therapy (42%), and hydrating infusive therapy or antidote drugs or extrabuccal feeding (8%).	268 restrained patients over study period.<br><br>Yearly admissions reported: 451 in 2008.	80/451 (18%) admitted patients restrained in 2008.	Most frequent reason “control of aggressive behavior” (81% of restraints of people with psychosis) compared with “prevention of damage or necessity of therapy”.	Duration of restraint 6 hr (SD 4.09) on average, the frequency of admissions with MRincreased across the 4 years (p = .006, chi-square), but mean restraints per patient and per admission not significantly different.<br><br>MR applied especially during the first 3 days of hospitalization, significantly less frequent during the morning and afternoon day shifts vs. night shift (p < .0001).<br><br>Restraints were more frequent during compulsory admissions or voluntary admissions of patients with altered state of consciousness.<br><br>Diagnosis of “schizophrenia and other psychotic disorders” was most frequent among the restrained patients, followed by the diagnosis of alcohol- and substance-related disorders. Voluntary admissions with restraints associated with significantly longer admission.
Dumais, 2011	2009	Canada	Retrospective audit of the use of seclusion and restraint in an adult psychiatric hospital 2007-2009.	Schizophrenia and other psychosis 44%, anxiety/depression 22%, bipolar disorder 12%, personality disorder 9%, substance-related 9%, mental retardation or other cognitive problems 5%.	Mean 49 (SD 17).	Mixed, male 54%.	Use of leather wrist and ankle restraints to keep patients in bed (applied only in the seclusion room).	23% (n = 632) were secluded with or without restraint at least once during the study period; most of those secluded (77%, n = 476) were subjected to restraint at least once.	2721	476/2721 (18%).	Patients may be secluded/restrained for: agitation, aggression, suicidal behaviours and self-harm, to protect them from harming themselves or others.	For those secluded and restrained, mean duration of restraint (in one or more episodes) was 22.4 h (SD 67 h), median of 5 h.<br><br>In bivariate analyses people restrained were more likely to be younger. Gender not a significant factor. Principal diagnosis of schizophrenia or other psychosis, bipolar disorder or personality disorder, and longer stay in hospital was associated with seclusion with restraint.
Eguchi, 2018	2014	Japan	Retrospective observational study using data from adult patients admitted to emergency or acute wards of a private psychiatric hospital.	All diagnosed with schizophrenia as per ICD-10.	Mean 41 (SD 12).	Mixed, male 44%.	MR using soft belts.	40% seclusion.	1559	114 (7%) both secluded and restrained.	Defined as emergency measure to limit behaviour and reactions for managing agitated or violent behaviours.	Seclusion with restraint resulted in favourable changes in psychosis/thinking disorder as measured by BPRS.

El-Abidi, 2021	2018	Spain	Descriptive study involving a sample of all patients admitted to two acute psychiatry hospitalization units.	Psychotic disorder 69%, depression 12%, substance abuse disorder 5%, others 15%.	Mean 42 (IQR 30-53).	Mixed, male 50%).	Immobilization through mechanical devices that cannot be easily controlled or removed.	-	474	129 (27%) restrained.	-	Median hours per episode 16 (IQR: 8-29, Z = 2.959), longer in men. In binary logistic regression, language barrier significant higher risk (OR = 2.13; 95%CI: 1.2-3.7; P = 0.007) and diagnosis also significant determinant: depressive disorder (OR = 0.22; 95%CI: 0.06-0.62; P = 0.005) and "other" diagnoses (OR 0.46; 95%CI: 0.23- 0.93; P = 0.03) vs. psychotic disorder.
Flammer, 2015	2014	Germany	Aggregated routine electronic data for 7 psychiatric inpatient units.	Main diagnosis as per ICD: FO/G3 8%, F1 31%, F2 17%, F3 24%, F4 13%, F5 0.3%, F6 6%, F9 2%.	Mean 46 (SD 19).	Mixed, male 52%	Use of belts to fix patient to the bed.	Seclusion in 579 admission (4%), involuntary medication in 78 admissions (0.5%).	15,832 admissions of 10,181 patients.	529 admissions (3%) involved MR (average 3.7 measures per affected admission).	-	Mean duration of MR 8.1 h (SD = 7.2).<br><br>Restraint in 228/14318 (2%) of voluntary admissions, 220/1026 (21%) of those detained under mental health legislation, 81/488 (17%) of those detained under guardianship legislation.
Flammer, 2020	2017	Germany	Central register data of 8 forensic hospitals (patients either preliminarily admitted awaiting trial following a crime, or subject to a hospital order).	Main diagnosis as per ICD: FO/G3 2.4%, F1 42%, F2 40%, F3 2%, F6 8%, F7 4%, F8 1%.	-	-	Physical restriction of movement by belts.	324 (23%) secluded, 9,358 seclusion episodes. Mean cumulated duration of seclusion episodes per affected case was 343.9 h (median = 90.8).	1431 patients	54 people (4%) restrained, 703 restraint episodes. Mean cumulated duration of restraint episodes was 261.7 h (median = 26.7).	-	Study compared with general psychiatric wards. The proportion of patients subjected to seclusion was about 8-fold higher than among patients in general psychiatric hospitals; proportion of patients subjected to MR was slightly lower.
Fugger, 2016	2012	Austria	Prospective study of all patients restrained in a psychiatric intensive care unit during study period. Ratings of subjective experiences were obtained at 4 study visits (including whilst retrained) where questioning was possible.	Of 47 restrained patients, as per ICD-10: n = 11 for F0, n = 6 for F1, n = 9 for F20.0, n = 4 for F20.2, n = 2 for F25.0, n = 7 for F31.2, n = 1 for F31.6, n = 1 for F33.3, n = 3 for F50.0, n = 3 for F60.3.	Mean 39 (SD 19) for restrained patients.	Mixed, 55% of restrained patients male.	Belt fixation.	Ward has no seclusion rooms.	216 patients were admitted, 120 (56%) involuntarily	47 (22%) were mechanically retrained.<br><br>Shortly after restraint, 39 (83%) patients were able to recall being restrained, 8 (17%) had no memory and were not included further.	-	On visual analogue scales, patients considered themselves depressed and powerless during restraint, with fear relatively absent. Anger was markedly present during physical restraint but not in consecutive visits as psychopathology improved.<br><br>Patients' acceptance of the coercive measure was significantly higher (P = 0.003), while patients' memory was significantly lower than expected (P < 0.001).<br><br>About 50% of the patients documented high perceived coercion, PTSD could be supposed in a quarter of the restrained individuals.
Fukasawa, 2018	2017	Japan	Centralised register data on admissions to general psychiatric wards (excluding forensic) in 113 wards from 23 institutions.	Total sample as per ICD: F0 9%, F1 6%, F2 35%, F3 28%,	-	Mixed, 46% male total sample.	5-point restraints to a bed or a chair on patient's arms, legs, and torso (fixing a patient at even one point is counted as restraint).	3679 (37%) at least one episode of seclusion (total example). Excluding older adult, 2715/7074 (38%).	7074 admissions excluding older adult.	Excluding older adult, 938/7074 (13%).	-	In admissions with at least on restraint, median total time under restraint 143 h. Both seclusion and restraint associated with longer mean duration of hospitalization, lower mean GAF score, more likely in wards with more beds, more nurses, in acute wards, and in urban areas, and restraint only more likely in organic mental disorders in addition to those diagnosed as having schizophrenia or related disorders. Adjusted odds ratio of the number of nurses per 10 beds for the use of restraint was 1.74 (95% CI, 1.35–2.24).
Georgieva, 2012	2009	The Netherlands	Prospective study at an acute adult inpatient psychiatric facility.	Psychotic disorder 20%, mood disorder 29%, personality disorder 19%, addition 28%, PTSD 4%.	Mean 40 (SD 13).	Mixed, 53% male.	Device which limits movement, physical activity, or normal access body	74 patients experienced coercion, of whom 46 (62%) were secluded only, 13 (18%) were involuntarily medicated, 12 (16%) were secluded and medicated.	518	3 patients (1%) were secluded and mechanically restrained.	-	Predictors were examined at level of all coercion rather than disaggregated.
Guzman-Parra, 2019	2017	Spain	Patients who had been subjected to coercive practice were assessed prior to discharge from two adult inpatient psychiatric units. Restraint compared to involuntary medication and combined coercion.	-	Mean 38 (SD 12).	Mixed, male 69%.	Application of mechanical fastening devices to limit physical mobility.	-	Total 111, of whom 32 subjected to MR, and 38 combined restraint and involuntary medication.	-	To prevent damage to the patient, other people, and/or the physical environment that surrounds them.	Significant differences in perceived coercion (CES) across groups (P < 0.001), higher in combined measures (M = 3.2, SD = 0.6), followed by MR (M = 2.8, SD = 0.8). Those who received combined measures (M = 20.9, SD = 15.6; P = 0.004) and MR (M = 21.5; SD = 14.9; P = 0.013) had higher scores in the DTS (Davidson Trauma Scale) compared to involuntary medication (M = 13.4; SD = 16.3). Those subject to combined measures were less satisfied with

												treatment (Client's Assessment of Treatment, CAT) than the group that received involuntary medication, however, the differences were not statistically significant when compared to the MR group.
Hilger, 2016	2013	Germany	Retrospective study of an inpatient clinic for patients suffering acute and chronic psychiatric disease, examining restraint and prophylaxis for venous thromboembolism (VTE) in prolonged restraint (>24 hours).	In prolonged restraint patients, 52% borderline personality disorder, 33% schizophrenia or schizoaffective disorder.	Mean age of prolonged restrained patients 47 (SD 16).	-	5-point fixation – both arms, both legs and trunk.	Did not include those who were secluded (numbers not reported).	12734 patients admitted	469 (7%) patients restrained. 36 (0.3%) were restrained longer than 24h.	-	None of the restraints (either prolonged, who are given VTE prophylaxis with enoxaparin, or lasting less than 24h, who are not given prophylaxis) were associated with clinical symptoms or signs of DVT.
Hirose, 2021	2017	Japan	Retrospective nested case control study using nationwide registers in Japan for case-control matching of patients with and without pulmonary embolism, to examine association with length of time in physical restraint in patients admitted to psychiatric departments.	In control (no pulmonary embolism): 34% schizophrenia, 33% mood disorder, 6% dementia, 27% other.	In controls median 51 (interquartile range 31).	Mixed, in controls 39% male.	As per mental health and welfare law in Japan, "restraint with a cloth or band specially made for restraint".	-	223,285 patients, 660 case-control pairs match by age and sex from same facility in same year were generated.	Overall, 13.2% patients received physical restraint (8% 1-14 days, 5% 15+ days).	-	Being in physical restraint for 15+ days was significantly associated with pulmonary embolism (OR 3.24, 95% CI 1.24-8.47).
Holm, 2021	2017	Denmark	Participants who had been exposed to MR during admission to the department of psychosis at a university hospital in the 5 years prior to the study, identified by registry data, were assessed using psychometric scales and interviewed regarding an index restraint episode.	75% paranoid schizophrenia or schizoaffective disorder, 15% bipolar disorder, undifferentiated schizophrenia or unspecified psychosis, 10% did not consent to diagnostic information.	Mean 37 (SD 10).	Mixed, 40% male.	"e.g. leather belts".	-	20 patients included. On average had experienced 19 MR episodes (SD 43).	-	-	On the Centrality of Event Scale, restraint episodes remembered at longer delays were rated more central to identity (non-significant, medium effect size); interpreting restraint episodes as central to identity was significantly related to higher PTSD symptoms (DSM-5 PTSD checklist), and centrality of episodes explained significant variation in PTSD symptom severity when controlling for trauma history and positive symptoms.
Hotzy, 2019	2016	Switzerland	Rates of restraint at three different acute adult inpatient psychiatric units in different states (NB attitudinal survey not otherwise included).	-	-	-	Use of restraining straps, belts or other equipment to restrict movement.	Zurich: 344 episodes of seclusion in 1699 admissions.<br><br>Muensingen: 632 episodes of seclusion in 2632 admissions.<br><br>Monthey: seclusion not used.	-	Zurich: 11 episodes of MR in 1699 admissions.<br><br>Muensingen: 203 episodes of MR in 2632 admissions.<br><br>Monthey: not used (but 17 episodes of physical restraint i.e. manual holds).	-	-
Hubner-Liebermann, 2005	1997	Germany & Japan	All inpatients with schizophrenia consecutively admitted to a psychiatric state hospital in Germany and a women's university psychiatric hospital in Japan.	All had ICD-10 schizophrenia diagnosis.	Germany: mean 39 (SD 14); Japan: mean 35 (SD 11).	Mixed.	"Straps around the belly or hands and feet".	-	856 patients in Germany, 50 patients in Japan.	7% of German sample, 20% of Japanese sample.	-	Samples did not differ in terms of suicidal behaviour and overt aggression during admission.
Husum, 2010	2006	Norway	Investigated coercive measures among patients admitted involuntarily to acute psychiatric wards, using linked ward data; included 32 wards across all 5 health regions in Norway.	Total sample 24% ICD-10 F20-29.	Total sample mean 40 (SD 16).	Mixed, total sample 50% male.	Strapping to bed with 5-point mechanical device, with belts over arms, legs and torso.	Patients exposed to any coercion ranged from 0-88% across wards.<br><br>424 secluded (35% of involuntary patients, 12% of total sample).<br><br>113 involuntary depot medication (9% of involuntary sample, 3% total sample).	Total sample 3462, of whom 1214 (35%) involuntarily admitted.	117 patients restrained (10% of involuntary patients, 3% of total sample).	-	In multi-level logistic regression, there was no substantial difference between male and female patients. Based on assessment of psychiatric problems with HoNOS, positive association between risk of restraint and aggressive/overactive (OR 2.38, 95% CI 1.91-2.98) and self-injury/suicidal symptoms (OR 1.39, 95% CI 1.12-1.73).<br><br>Patients from ethnic groups other than Norwegian had a lower risk of restraint (OR 0.39, 95% CI 0.16-0.96).<br><br>Wards in urban area significantly associated with restraint (OR 3.58, 95% CI 1.28-4.86).

Keski-Valkama, 2007<br>&<br>Keski-Valkama, 2010<br>&<br>Keski-Valkama, 2010	2004	Finland	Structured postal survey of Finnish psychiatric hospitals for adults of working age, during predetermined weeks in 1990, 1991, 1994, 1998 and 2004, examining details of the first restraint/seclusion incident of each patient in the study week, combined with register data. Study period was characterised by legislative changes resulting in more restrictive and specified regulations in the use of coercion.	Among the 671 restrained/ secluded patients, psychosis 64%, substance-use related 10%, affective disorder 8%, personality disorder 2%.	In restrained/ secluded patients mean 39 (SD 11).	Mixed, 56% of restrained/ secluded patients male.	Confining patient to a restraint bed.	In 2004, 59/4589 (1%) secluded.	Total population across survey years 28064 (range 4589-6417 in individual years).	In 2004, 36/4589 patients (1%) restrained.	Indication for restraint: actual violence 20%, threatening violence 11%, damaging property 6%, agitation/disorientation 56%, aggression/ dangerousness 4%.	Significant regional variation.<br>Risk of restraint decreased in 2004 vs 1990 RR 0.67 (95% CI 0.45-.99), but no linear trend formed.<br>Duration of restraint incidents did not change over study period. Median duration 425min (30-3045) in 1991, 420 in 2004 (30-3705).
Knutzen, 2007	1997	Norway	Retrospective study using routine electronic data from an acute psychiatric ward, of all patients admitted during 2 years.	-	-	Mixed, male 48%.	Belts, for restraint in a bed or for arms and feet, or straitjackets.	Pharmacological restraint only in 2%. No seclusions.	960 admitted patients.	48 (5%) MR only, and 67 (7%) both pharmacological and MR.	-	Analyses at level of all restraint.
Knutzen, 2013<br>&<br>Knutzen, 2014	2005	Norway	Included all restrained patients admitted to the acute psychiatric wards of three Norwegian hospitals during a 2-year study period, using routinely collected data.	Of patients mechanically restrained, F20-29 35%, F10-19 19%, F32-39/40-49 7%, F30-31 15%, F60-69 11%.	18-29: 32%, 30-39: 27%, 40-49: 21%, 50+: 20%.	Mixed, male 56%.	Belts, for restraint in a bed or for arms and feet.	Of restrained patients, 18% pharmacological restraint only.	371 restrained patients.	MR was the most prevalent: 83% mechanically restrained, 35% with concurrent pharmacological restraint.	87% of MR was for assault.	Most (56%) were restrained only once, 35% 2-5 times, 9% 6+ times (who accounted for 39% of all restraint episodes).<br>Male patients were mechanically restrained (+/- pharmacological) significantly longer than women. More males were restrained >30h (10% vs 4%) and fewer <3h (23% vs. 43%).<br>Patients mechanically restrained (+/- pharmacological) for self-injury were restrained significantly shorter periods than for other reasons.<br>None of the variables (age, immigrant or native born, voluntary or involuntary status, diagnosis, length of stay or number of admissions) were associated with duration of MR.
Kodal, 2018	2014	Denmark	Descriptive study of admissions to a mood disorder unit consisting of 4 wards, using data from electronic reporting system combine with ward-level data such as staffing rotas, over 1 year study period.	Of restrained patients, 82% personality disorders, 10% mood disorders.	Of restrained patients, mean 29 (SD 6).	Of restrained patients, mixed, 10% male.	Enforced fixation to bed by use of leather belt around the waist.	-	259 admissions (not reported how many unique patients).	Restraint in 20 patients (114 times).	-	Three individuals accounted for 77% of restraints.<br>Median duration of restraint 9.7h (range 0.2-330.1h).<br>In multiple regression analyses, evening shift (OR 1.29, 95% CI 1.14-2.57), months with January as baseline (OR 0.88, 95% CI 0.83-0.94) and male gender of care workers (OR 1.44, 95% CI 1.01-2.05) were significantly related to restraint.<br>No associations were found between restraint and staffing level, age, education, experience of care workers or change of shifts.
Kostrecka, 1999	1987 AND 1996	Poland	Comparison of two periods pre and post 1995 Polish Mental Health Act in 11 locked wards in Warsaw.	1989: n=98 restrained: 54% percent endogenous psychoses, mainly schizophrenia. alcohol dependence, 23%. 1996 N= 65 restrained: 74% schizophrenia, 11% alcohol dependence.	Mean 43.	1989 n= 45% male.<br>1996, n= 60% male.	Four-point leather restraint, a camisole, or restraining sheets.	-	452 in 1989<br>414 in 1996.	1989: 21%<br>1996: 15%.	Aggression: 61% in 1989, 74% in 1996	No associations between demographic or diagnostic variable or any variables related to physical conditions on the ward (including male/female).<br>Duration of restraint longer when more patients per staff (r=.7, p=.02).
Lau, 2020	2018	Switzerland	Longitudinal, observational dynamic cohort study (tracked data in a single forensic psychiatric institution): 9-year follow-up data (2010–2018).	90% schizophrenia, of others, 90% substance misuse as secondary diagnosis.	-	Varying each year, range: Male 85-90%, Female 10-15%.	Device used to fixate a patient (*e.g.*, a belt).	In 2018, 19% patients secluded, 9% forcibly medicated.	Varying each year, range 118-125.	Ranged from 4 to 14 (3% to 11% for the given year). In 2018 9/123 patients (7%)	-	Wide variation in max and median periods in restraint.

Laukkannen, 2019	2017	Finland	Register data for the year 2017 collected directly from 140 inpatient psychiatric wards within 21 organizations.	-	-	-	Equipment, such as belts, to limit movement.	-	-	MR was used 2,113 times across 106 wards in 2017:<br><br>38.83 per 100,000 inhabitants, mean duration 0.7 days.	Not reported.	-
Leerbeck, 2017<br><br>&<br><br>Linkhorst, 2022<br><br>&<br><br>Martensson, 2019	2012<br><br>&<br><br>2016<br><br>&<br><br>2014	Denmark	Register-based, cross-sectional study of patients aged ≥ 18 years admitted to psychiatric wards who had been subjected to coercive measures in three different overlapping periods across the three papers, 2010-2012, 2011-2016 and 2010-2014.	-	Median 46, interquartile range 31-59 years for women and 42 (30-56) for men.	Mixed, 54% male.	Use of belt to fixate patients to bed, further restraining movements of hands and feet by using straps, further restraining use of fingers by using gloves.	-	-	In 2010-2012, 5,456 episodes of MR among 7,338 unique persons.	A total of 236 first MRs and 149 cases of door locking were requested by the patient.	Men were more often subjected to MR.<br><br>Patients with dual diagnosis were more often mechanically restrained compared to patients with only psychiatric diagnoses or only other substance use diagnoses, attenuated when the characteristics of patients accounted for.<br><br>Patients with only other substance related diagnoses had the highest risk of being mechanically restrained.<br><br>MR was the most common first coercive method and initiated more often in evening than in day shifts.
Lorenzo, 2014	2012	Italy	Retrospective cohort study conducted in a 15-bed public psychiatric ward, cumulative data over 7 years (2005-2012).	Schizophrenia and other psychoses (32%), organic psychosis (20%), bipolar disorder (14%).		62% male.	Wrist restraints for both upper and lower limbs, which allow the immobilization of the patient on the bed in the supine position (in some cases abdominal belt of restraint is applied).		305 restrained patients (compared to non-restrained patients in reporting statistics).	1224 episodes; 4 times per patient on average; duration of 6 hours on average	To control aggressive behavior 64%, prevention of injury/urgent therapy 36%.	More frequently applied during the night shifts and in the first days of hospitalization. Average length of hospitalizations with restraint was higher than other hospitalizations<br><br>Female gender (p<.001), diagnosis of "schizophrenia and other psychoses" (p<.005), "bipolar disorder" (p<.05), "neurotic disorders" (p<.01) and "personality disorders" (p<.05) were protective.<br><br>Extra-European nationality (p<.001), "organic psychoses", as second and third psychiatric diagnosis (p<.01, p<.001), organic comorbidity (p<.001), compulsory state of hospitalization (p<.001), and long duration of hospitalization (p<.001) were risk factors.<br><br>Replacement of medical staff (p<.001) and the implementation of more restricted guidelines for restraint application (p<.001) were protective.<br><br>In hospitalizations with restraint, organic comorbidity and compulsory admission were more frequent.
Lykee, 2019	2012	Denmark	Patients affected by severe mental illness and comorbid substance abuse that were hospitalized in 3 large wards (single hospital), 2006-2012.	Substance misuse disorder plus: schizophrenia spectrum disorder (50%) or personality disorder (20%).	Mean 40.	70% male.	Fixation by a mechanical device, which includes immobilization with leather belts.		1698 hospitalisations.	MR ranged between 1% and 4% per year. Overall 35/1698 people admitted over study were restrained (2%).	Aggression/threatening behavior (41%), extreme agitated state (32%), physical violence toward staff or personnel (15%), destruction of property and endangering self or others (12%).	MR predominantly implemented during day (8am-4pm) and evening (4pm-12am) shifts (82%). MR only administered 18% of the time in early morning (12am-8am) when staff–patient ratios lowest.<br><br>Schizophrenia (OR 2.64; 95% CI 1.29-5.40), use of stimulants (OR 5.68; 2.78-11.59) and male sex (OR 3.22; 1.12-9.27) were positively associated with MR.<br><br>Those exposed to MR had significantly longer hospitalizations vs. those not exposed to MR (8.3 vs 3.5 months).
Mann, 2021	2014	Germany	Multicenter prospective observational study, enrolled patients in 4 psychiatric hospitals located in the German federal state of Rhineland-Palatinate over a period of 24 months (2012-2014).	Substance-related disorders (F1) were most frequent, followed by organic mental disorders (F0) and schizophrenia and other psychotic disorders (F2).	-	65% male.	MR in a bed or chair.	-	Seclusion rare (0-5%).	1542 cases of coercive measures: 85-100% of these were MR across the 4 hospitals.	-	MR and seclusion examined together however as seclusion was rare, data presented here.<br><br>ORs did not change considerably after adjusting for sex, diagnosis, and occupancy per staff.<br><br>In younger age group, diagnosis of substance use disorder (ICD-10: F1) was associated with a lower probability of a cumulative duration >8h vs. other diagnoses, and male patients more likely to experience a cumulative duration >8h vs. female.

Martin, 2007	2004	Germany & Switzerland	Comparative observational study of 7 Swiss and 7 German psychiatric hospitals in 2004.	Of patients in Swiss sample, 25% had a schizophrenic disorder (F2).<br><br>In German sample, schizophrenic disorder in 22%.	-	-	Use of belts to fix a patient to a bed.	Seclusion in 18% of Swiss sample and 8% of German sample.<br><br>In the Swiss hospitals 1% were affected by seclusion as well as MR and 23% exposed to at least one kind of intervention (MR or seclusion). In the German hospitals equivalent figures were 2% and 16%.	-	In Swiss sample, 130/1976 admissions affected by MR (7%) (range among hospitals 1-14%)<br><br>In German sample, 700/6761 admissions affected by MR (10%) (range among hospitals 7-16%).	-	-
McKenna, 2017	2014	Australia	Retrospective cohort across Victoria's 21 state-funded adult area mental health services which included people subjected to prolonged restraint (>1h) or prolonged seclusion (>8h).	-	Among those subjected to prolonged MR: age range 16–63 years, mean 35 years.	Mixed, male 66% among those subject to prolonged MR.	Victorian mental health legislation defines as use of a device to prevent or restrict movement.		311 patients subjected to prolonged restrictive intervention (131 subject to prolonged MR).	690 episodes of prolonged restrictive intervention, of which 320 (46%) were MR.	Risk of harm to others 88%, symptom management 53%, planned 39%, absconding risk 20%.	Compared to seclusion, MR was more often planned with the patient and more likely to have involved a medical transfer offsite or to another unit, or the person assessed as an absconding risk.<br><br>Median duration significantly longer for males, those aged ≥35 years, episodes in adult area mental health services, and for those who were subject to multiple episodes of prolonged MR.<br><br>Some patients experienced multiple episodes of pro-longed MR (2–4 episodes: 21%(n=28), ≥5 episodes: 2% (n=3)). Median duration of 320 episodes of prolonged MR 4h.
Muller, 2023	2020	Switzerland	Observational study using clinical, procedural, and sociodemographic data from patients treated as inpatients in Switzerland's largest psychiatric institution during the years 2017-2020.	Substance use disorders 27%, psychotic disorders 24%, depression 21%.	Mean 39.9.	Male 56%.	Strapping the patient to a bed with belts with 5-point restraints (arms, legs, and torso) or less.		8,700 adult patients with 16,607 admissions.	MR in 0.3% (n = 44) admissions.	Other data at level of pooled coercive measures.	-
Newton-Howes, 2020	2019	Australia, New Zealand, Japan and the United States	Epidemiological study of MR using country-wide, publicly available data for Pacific Rim countries, for patients admitted to adult mental health hospitals or mental health wards of a general hospital. Attempted to exclude forensic, child, geriatric and learning disability wards. Countries compared in 2017, and time trends 2003-2019.	-	-	-	Using a device of any sort, other than restraint by third persons delivering a patient to the ward (e.g. the police bringing someone into the ward in handcuffs).	-	-	Mean number of restraints per 1000 bed days in 2017: Australia 0.92, New Zealand 0.14, US 1.48, Japan aged 20-64 37.4.	-	Large variations within and between countries and over time.<br><br>Despite international reduction goals, the only age group for which restraints were reduced in all countries over time, was the >65-year-old group after 2016 or 2017.
Noda, 2013	2009	Japan	Observational study using medical records of patents admitted to three emergency wards and three acute wards at four psychiatric hospitals during 8-month study period.	In patients who were secluded or restrained, as per ICD: F2 59%, F3 15%, F1 8%, F0 7%.	Mean 46 (SD 17).	Mixed, male 52%.	Use of restraining straps, belts, or other equipment to restrict movement.	687 secluded (56%).	1232 admitted patients.	148 patients restrained (12%).	Reported at level of seclusion or restraint.	Mean duration restraint 142.0 ± 230.4 h and the median (IQR) was 82h (29–159h), with the highest prevalence occurring between 24 and 48h.<br><br>Duration of restraint was significantly longer for men than for women, and longer for patients with F20–F29 diagnosis than for those with F10–F19 diagnosis.
Noorthoorn, 2015	2011	The Netherlands	Observational study using data from hospitals where the Dutch mental health act applies. Included 20 mental health institutes and 3 psychiatric departments of general hospitals covering 75 hospital locations and 375 wards. Covered around 75% of all admissions.	Schizophrenia 32%, drug abuse 26%, personality disorders 26%, mood disorders 23%, organic disorders 2.6%, neurotic 15%, mental handicap 2.6%, childhood onset 5%. Developmental disorder 5%.	-	-	Use of belts to fix patient to a bed or chair.	4725/42960 (11%) experienced seclusion. 69 (0.2%) experience both MR and seclusion. 41 (0.1%) experienced MR, seclusion and involuntary medication.	42960	379/42960 (1%) experienced MR.	-	2.7 patients exposed to MR per 100,000 inhabitants (2.5 excluding forensic patients), primarily on geriatric wards or forensic wards.<br><br>Most prevalent diagnosis in MR group was an organic mental disorder, followed by personality disorder.<br><br>MR associated with older age (OR for young age 0.19, 95% CI 0.07-0.46) and childhood onset disorders (OR 3.75, 95% CI 1.5-9.3).<br><br>Inverse association with patients of ethnic minority (OR 0.24, 95% CI 0.07-0.9).

Porat, 1997	1992	Israel	Survey of all hospitalised patients in government-owned psychiatric hospital closed wards who underwent restraint during a one-month period, using restraints register data and ward nursing records.	Of restrained patients, 87% psychotic.	Of restrained patients, <19, 6%; 20-34, 44%; 35-49, 32%; 50+, 18%.	Mixed, 66% male in restrained patients.	Securing patient to a frame, usually a bed, by 2 or 4 limbs. In study, 91% involved securing by 4 limbs to a special bed.	The facilities had almost no facilities for seclusion.	1419 admitted patients.	202 (14%) restrained.<br>Ranged from 8% to 37% across individual hospital sites.	Agitated behaviour 37%, violence 32%, aggression 20%, danger to patient 6%, to permit treatment 3%.	Non-psychotic patients were held longer in restraint than psychotic patients.<br>Those on acute wards were held longer in restraint than long-stay wards.<br>Peak of restraints at 10am and 9pm.<br>Those restrained in the 3pm-9pm shift restrained 25% longer than those restrained during day shift.
Reitan, 2018	2011	Norway	Observational study of use of restraint in adult acute psychiatric wards over 8-year period 2004-2011. Data collected retrospectively from handwritten protocols. Collected data on all people mechanically or pharmacologically restrained.	Of patients either mechanically or pharmacologically restrained, organic mental disorder 10%, psychoactive abuse 14%, schizophrenia spectrum disorder 37%, mood disorders 21%, personality disorder 5%.	Of cases of MR, 37% 18-29, 20% 30-39, 43% 40-49, 13% >50.	Mixed, 55% of cases of MR male.	Straps on limbs and/or chest binding the patient to a bed or straps used to minimize movement during walk.	41% of cases of restraint were pharmacological.<br>Seclusion was not used as restraint, and holding was not registered until 2008.	19283 patients admitted over study period (of whom 324 were subjected to either mechanical or pharmacological restraint).	863 instances of MR over study period (59% of cases of restraint were MR).	-	Use and type of restraint varied significantly by season (p = 0.043). During summer, MR used significantly more often than pharmacological restraint, vice versa in spring.<br>Significant diurnal variation (p = 0.007), more between 12:01 and 00:00.<br>Once subjected to restraint, males would significantly more often be subjected to MR compared to females.<br>MR was more often used for younger patients, and pharmacological restraint for older.<br>Once subjected to restraint, risk of MR rather than pharmacological restraint significantly higher in organic mental disorder (p = 0.005).
Staggs, 2015	2013	United States	Study using administrative data to examine trends in seclusion and restraint in response to injurious assaults by patients on adult psychiatric units 2007-2013. Comprised 438 inpatient units in 317 US hospitals. Units serving non-adult populations, or with a behavioral health or specialty (dual diagnoses) designation were excluded.	-	Men age of assaultive patients 39 (SD 17).	Mixed, males accounted for 57% of assaults.	Devices, e.g. blanket wraps, net restraints, and four-or five-point restraints.	930/8002 (12%) assaults responded to with pharmacological restraint.<br>651/8002 (8%) responded to with holds.<br>1362/8002 (17%) responded to with seclusion.	8002 injurious assaults.	1449 (18%) of injurious assaults responded to with MR.	-	There was a statistically significant decreasing trend in the percentage of restraints involving devices over the study period.<br>There was little evidence suggesting a decline in the use of seclusion or restraint during the study period.
Tarsitani, 2013	2010	Italy	Prospective study to examine restraint amongst first generation immigrant patients admitted to adult psychiatric intensive care unit of an academic hospital, vs. Italian-born patients, 2008-2010. Excluded patients previously admitted to the PICU.	Psychotic disorder 51%, mood disorder 42%, other 7%. Excluded those with severe medical illness, cognitive impairment, delirium or substance-induced disorder.	Mean 36 (SD 11).	Mixed, male 42%.	Physical restraint by belt.	-	100 immigrants and 100 non-immigrants matched by age, gender and diagnosis.	Restraint by belt used at least once in 7% of all included patients.	Restraint is strongly discouraged and only used when necessary for the containment or the prevention of actual violent behaviour.	Immigrant patients more likely to be restrained vs. Italian-born patients (11% vs 3%; $\chi^2 = 4.92$; p = 0.027; RR=3.67; 95% CI = 1.05–12.7). No significant differences between groups in rates of repeated restraints or overall duration of restraint.
Tavcar, 2005	1999	Slovenia	Naturalistic observational study of a Psychiatric Intensive Care Unit where net-beds had been used for decades but were abandoned in 1999 in favour of bed-belts. Data from the charts of all patients admitted during two two-month periods in 1998 and 1999.	-	-	Mixed, in 1998 52% male, in 1999 48% male.	Net beds and bed-belts.	-	332 patients admitted in 1998 sample, 312 in 1999.	In 1998, coercive physical measures were used with 32 patients (10%); 24 (7%) were managed with net-beds, and eight (2%) with bed-belts. In 1999, coercive physical measures were used with 16 patients (5%); all bed-belts.	Violence was the most cited reason for use of bed-belts and confusion (delirium) for use of net-beds.	Significantly more patients with schizophrenia were managed with bed-belts.<br>In 1999 fewer coercive physical interventions were used. Use of bed-belts reduced, reserved for most severely disturbed patients. As other characteristics not change, authors concluded changes in availability of certain coercive measures explained reduction.<br>Patients managed with bed-belts had significantly higher scores on CGI than those managed with net-beds (5.6 vs. 4.9; p<.05); lower GAS scores (18 vs 31; p<.01) and higher BPRS scores (65.3 vs. 53.9; p<.05).
Valimaki, 2019	2014	Finland	Nationwide registry study of adult patients admitted to psychiatric units (excluding forensic), examining use of coercive measures over a 20-year period 1995-2014.	Any primary psychiatric diagnosis according to ICD-9 or 1CD-10 classifications.	Mean 44 (SD 16).	Mixed, male 52%.	Limb restraint, when a patient may be tied down with belts or comparable tools.	Seclusion 7%, forced injection 3%, physical restraints (holding) 1%.	226948 patients admitted during 505169 hospital periods.	Overall 19113 (4%).<br>In 2010-2014, 3162/108345 (3%).	-	Only the use of limb restraints showed a downward trend over time, starting in 2000-2004. Consistent decline from its peak at 5% in 2001 to 3% in 2014.<br>Significantly lower prevalence in females.

Valimaki, 2022	2018	China (Hong Kong)	Register-based cohort study of 14 wards in two psychiatric hospitals (closed wards, either acute admission wards, psychiatric intensive care units or rehabilitation wards).	Majority schizophrenia and mood disorders. Breakdown not reported at total population, in restrained patients: 61% psychotic disorders, 18% neurodevelopmental disorders, 13% mood disorders.	18-65 years. Mean of restrained patients 40 (SD 16).	Mixed, not reported at total population, 50% male in restrained patients.	Device to limit or prevent movement of the whole or a portion of the patient's body e.g. safety vest, waist belt, limb holder, magnetic shoulder trap.		4170 patients admitted during study period.	"Rate of patient restraint events was 0.43".	Violent behaviour (47%), self-harm (6%) and absconding (5%).	The most critical time points for physical restraints were late afternoon (17:00h-18:00h) and later in the evening (20:00h - 21:00h).<br><br>About half of all restraints took 1–2h (52%), and seldom lasted less than one hour (5%). Only two lasted >4h. Longer if a restrained person was younger, male, not treated on a voluntary basis, and diagnosed with a neurodevelopmental disorder.
van Heesch, 2022	2020	Belgium	Study of coercive measures in a high security Forensic Psychiatric Center (FPC) in Belgium, including all patients admitted over a 6-year period 2014-2020. 83% of patients had a violent index offence, and almost all (99%) were in prison prior to admission.	Primary diagnosis psychotic disorder 36%, personality disorder 35%, paraphilic disorder 14%, other 16%.	Mean 42 (SD 12).	Predominantly male (98%).	Any external mechanical devices for limiting the patients' movement.	Seclusion in 48%, chemical restraint 12%.	654 patients admitted	5 (1%) patients were mechanically restrained (7 episodes).<br><br>Note: in the Flemish FPCs, there is a non-MR policy with no restrictive devices standardly available.	One disoriented patient with Parkinson's disease was fixated in bed at night to avoid falling, another patient was shackled and three patients were fixated on their wrists to prevent self-harm.	Female patients (n = 15) were not subjected to MR.
Visaggio, 2018		United States	Retrospective chart review study of three adult psychiatric institutions including all cases where a patient was restrained or secluded.	Discharge diagnosis schizoaffective disorder 37%, mood disorders 16%, bipolar disorder 15%.	Mean 32 (SD 13).	Mixed, male 48%.	4-point MRs or a restraint chair	-	743 cases of MR, including 332 cases of restraint chair and 101 cases of 4-point MR.	-	Patients who were restrained to administer court ordered treatments were excluded.	Out of the 332 episodes involving the restraint chair, 5 (2%) had patient injuries and 13 (4%) had staff injuries. Among the 101 episodes of 4-point MR, 3 (3%) involved patient injuries and 9 (9%) involved staff injuries (4-point restraints significantly increased risk of staff injury vs. both other methods).<br><br>For the 310 episodes of seclusion, 11 (4%) had patient injuries and 6 (2%) had staff injuries.
Wu, 2015	2014	China (Hong Kong)	Retrospective observational study of patients admitted to the acute psychiatric ward of a public hospital. Recruited with a convenience sample and medical records used to classify into restrained and non-restrained group.	Restraint group: schizophrenia or schizoaffective disorder 27%, paranoid schizophrenia 12%, bipolar disorder 11%, acute psychosis 8%, personality disorder 8%, drug-induced psychosis 9%, depression 8%, mental retardation 9%, dementia 2%, delusional disorder 1%.	Restraint group: 38 (SD 15), non-restraint 44 (SD 17).	Mixed, restraint group 42% male, non-restraint group 44% male.	Safety vests, magnetic limb holders/shoulder straps, pelvic holders, magnetic waist and abdominal belts applied to wrists, ankles, shoulders, waist and body to restrict freedom of movement or secure to bed or chair.	-	335	133/335 (40%) were restrained in the first 7 days of admission.	-	5 factors emerged as significant predictors: involuntary admission (OR 4.4), current use of psychiatric medications (OR 0.19), no history of violent behaviour (OR 0.33), age (OR 0.98) and depression (OR 0.43).
Zhu, 2014	2012	China	Study of all consecutively admission patients to an adult psychiatric ward who were able to consent.	Schizophrenia 57%, mood disorders 28%, others 15%.	Mean 30 (SD 12).	Mixed, 49% male.	Use of belts to fix a patient to a bed.	-	160	82 (51%) were restrained.	-	Compared with non-restrained patients, restrained patients were younger (OR 0.97, 95% CI 0.93-0.99), had more aggressive behavior before admission (OR 1.1, 1.04-1.2), received less outpatient psychiatric treatment before admission (OR 0.3, 0.1-0.9), and were more likely to receive mood stabilizers (OR 5.6, 95% CI 1.9-16.7). Male patients were less likely to be restrained (OR 0.2, 0.1-0.6).

CGI, Clinical Global Impression; BPRS, Brief Psychiatric Rating Scale; PTSD, Post-Traumatic Stress Disorder; GAF, Global Assessment of Functioning; CES, Coercion Experience Scale; GAS, Global Assessment Scale.

Supplement 5

Quality assessment for studies reporting prevalence or rate of use of mechanical restraint

We used the quality assessment checklist for prevalence studies(30) adapted from Hoy et al.(31) Total score was converted to a summary risk of bias of low (0-3), moderate (4-6) and high (7-9).

For item 1, if sex was withing 60:40/40:60 ratio, and population average age 30-50, this was interpreted as within normal range. For items 2 and 3, where entire populations (e.g. of hospital inpatient registers) were included, this was interpreted as a 'census' being taken. So, of the population being studied, there was little or no risk of sampling bias. For item 5, where data were taken directly from subject records (which was appropriate), this was scored as 0. For item 7, any forms designed for the purposes of the study, though having face validity, were not considered to be 'validated and reliable' as we are not aware of any tool that exists for the purposes of this research.

Study	RoB checklist item score									Total	Overall RoB
	1	2	3	4	5	6	7	8	9		
Andersen, 2016	1	0	0	0	0	0	1	0	0	2	Low
Bak, 2014 & Bak, 2015	1	1	1	0	1	0	1	0	0	5	Moderate
Bilanakis, 2010	0	0	0	0	0	0	1	0	0	1	Low
Chieze, 2021	0	0	0	0	0	0	1	0	0	1	Low
Danielsen, 2019	0	0	0	0	0	0	1	0	0	1	Low
Dazzi, 2017	0	0	0	0	0	0	1	0	0	1	Low
De Hert, 2010	1	1	1	0	0	0	1	0	0	4	Moderate
Di Lorenzo, 2012	0	0	0	0	1	0	1	0	0	2	Low
Dumais, 2011	0	0	0	0	0	0	1	0	0	1	Low
Eguchi, 2018	1	1	1	0	0	0	1	0	0	4	Moderate
El-Abidi, 2021	0	0	0	0	0	0	1	0	0	1	Low
Flammer, 2015	0	0	0	0	0	0	1	0	0	1	Low
Flammer, 2020	1	0	0	0	0	0	1	0	0	2	Low
Fugger, 2016	0	0	0	0	0	0	1	0	0	1	Low
Fukasawa, 2018	0	0	0	0	0	0	1	0	0	1	Low
Georgieva, 2012	0	1	0	0	1	0	1	0	0	3	Low
Hilger, 2016	0	0	0	0	0	0	1	0	0	1	Low
Hirose, 2021	1	0	0	0	0	0	1	0	0	2	Low

Hotzy, 2019	1	1	1	0	0	0	1	0	1	5	Moderate
Hubner-Liebermann, 2005	1	1	0	0	0	0	1	0	0	3	Low
Husum, 2010	0	0	0	0	0	0	1	0	0	1	Low
Keski-Valkama, 2007<br>&<br>Keski-Valkama, 2010<br>&<br>Keski-Valkama, 2010	0	0	0	0	0	0	1	0	0	1	Low
Knutzen, 2007	0	0	0	0	0	0	1	0	0	1	Low
Kostrecka, 1999	0	0	0	0	0	0	1	0	0	1	Low
Lau, 2020	1	0	0	0	0	0	1	0	0	2	Low
Laukkannen, 2019	1	0	0	0	0	0	1	0	0	2	Low
Leerbeck, 2017<br>&<br>Linkhorst, 2022<br>&<br>Martensson, 2019	0	0	0	0	0	0	1	0	0	1	Low
Lorenzo, 2014	1	0	0	0	0	0	1	0	0	2	Low
Lykee, 2019	1	0	0	0	0	0	1	0	0	2	Low
Mann, 2021	1	0	0	0	0	0	1	0	0	2	Low
Martin, 2007	1	0	0	0	0	0	1	0	0	2	Low
McKenna, 2017	1	0	1	1	0	0	1	0	0	4	Moderate
Muller, 2023	0	0	0	0	0	0	1	0	0	1	Low
Newton-Howes, 2020	0	0	1	0	0	0	1	0	0	2	Low
Noda, 2013	1	0	0	0	0	0	1	0	0	1	Low
Noorthoorn, 2015	1	0	0	0	0	0	1	0	0	0	Low
Porat, 1997	0	0	0	0	0	0	1	0	0	1	Low
Reitan, 2018	0	0	0	0	0	1	1	0	0	2	Low
Staggs, 2015	0	0	0	0	0	0	1	0	0	2	Low
Tarsitani, 2013	0	0	0	0	0	0	1	0	0	1	Low
Tavcar, 2005	0	0	0	1	0	0	0	1	0	2	Low
Valimaki, 2019	0	0	0	0	0	0	0	1	0	1	Low
Valimaki, 2022	0	1	0	0	0	0	0	1	0	2	Low

van Heesch, 2022	0	1	0	0	0	0	0	1	0	2	Low
Wu, 2015	0	0	0	0	0	0	0	1	0	1	Low
Zhu, 2014	0	1	0	0	0	0	0	1	0	2	Low

Supplement 6

Quantitative studies of interventions to reduce restrictive practice and/or impact of ceasing restraint on other outcomes. Empty cells marked with (-) indicate information not reported.

Study Ref	Last year data collected	Country	Study details, population, setting	Change or intervention	Diagnoses	Age	Sex	Restraint device	Prevalence of other forms of coercion	Total N of population examined	Prevalence (%) or rate of MR	Indications for MR	Impact of change and other patterns or associations
An, 2016	2013	China	Consecutively admitted patients to an adult teaching psychiatric hospital able to give consent, before and after implementation of National Mental Health Law (NMHL).	Implementation of National Mental Health Law, which emphasized the protection of psychiatric patients' rights, dignity and interests.	Schizophrenia 33%, mood disorders 43%, other 24%.	Mean 36 (SD 14).	Mixed, male 36%.	Immobilisation with a mechanical device	-	1364	31% of 789 recruited pre-NMHL implementation, 22% of 575 recruited post-NMHL implementation were restrained.	If "the potentially dangerous behaviour was the consequence of a psychiatric disorder…to protect the patient and/or others' safety, when the patient has refused the necessary treatment in an emergency, such as violence or suicide attempt."	Multiple logistic regression analysis revealed that restraint was independently associated with unemployment, lower income, aggression in the past month, being admitted before the NMHL implementation and having poorer insight.
Andersen, 2017	2015	Denmark	Comparative study of two psychiatric units, where one implemented sensory modulation to reduce seclusion/restraint and one unit served as the control group.	Sensory modulation. Staff had weekly supervision by a skilled occupational therapist. Study unit also had access to a variety of sensory modalities and a sensory room.	Various psychiatric disorders such as schizophrenia, bipolar disorder, and depression.	18-65.	Mixed, proportion not reported.	Belt restraint	-	5371 bed days in intervention group, 4627 in control.	-	-	18 restraints per 5371 bed days (rate 0.0033) in intervention, 25 per 4627 in control (0.0054) (non-significant).
Badouin, 2023	2022	Germany	Pre–post study of implementation of peer support in one locked ward compared to treatment as usual in a second locked ward of a psychiatry department.	Peer support, part of the "PACT" study ("Peer Supported Autonomy-Promoting Crisis Treatment").	Schizophrenia (47% intervention, 41% control), substance abuse (27%, 39%), affective disorders (7%, 9%).	39 (SD 15) in intervention, 39 (12) in control (all figures post-assessment).	Mixed, 62% male in intervention group, 65% male in control.	Fixation via wrist and ankle cuffs attached to the patient's bed	Overall MR accounted for 91% of all restraint episodes. Combined MR and forced medication (8%). Forced medication alone 1%	373 (in post-intervention analyses).	40/200 (20%) exposed to MR in intervention group in post-assessment period; 46/173 (27%) in control group.	Situations in which no other means insufficient to prevent further harm; pose a critical threat to the patient's or others' well-being. Statutory regulations stipulate patient must demonstrate an inability to exercise self-determination.	No change in the proportion of patients experiencing restraint in the intervention group (increased in control group). Note, study disrupted by COVID-19.
Celofiga, 2022	2019	Slovenia	Cluster randomised trial of all acute wards of psychiatric hospitals (6 hospitals, 12 wards) in Slovenia to assess the effect of de-escalation training.	Training in verbal and non-verbal de-escalation, according to the Beta Project of the American Association for Emergency Psychiatry, the World Health Organization Quality Rights training to act, unite and empower for mental health, and a skills handbook by Amdur et al.	ICD: F0 13%; F1 22%; F2 34%; F3 18%; F6 2%; Other 11%.	Mean 48 (SD 17) in intervention group, 49 (18) in control.	Mixed, 54% male in intervention. group, 53% in control.	Restraint with belts	-	6,401 patients (3,190 in baseline period, 3,211 in intervention period). NB patients in study = treatment episodes.	5% of patients were restrained due to aggression in the intervention group vs. 10% in the control (no differences at baseline, 9.9% and 8.7%). 3% of intervention group and 2% of control group restrained for reasons other than aggression.	-	During the intervention period, incidence rate of restraints due to aggression in the experimental group decreased to 30% of the rate in the control group (IRR = 0.304, 95% CI 0.24; 0.39]). No reduction in the incidence of restraint used for reasons unrelated to aggression.

Flammer, 2021<br>&<br>Flammer, 2022<br>&<br>Flammer, 2021b	2020	Germany	Central register data from 31 adult psychiatric hospitals (excluding forensic), to examine predictors (2015-2017), changes in use between 2017 and 2018 after introduction of "immediate judge's decisions" in 2018, and 2019-2020 to examine impact of COVID-19. In 2020, 14% of cases involuntary admissions.	Introduction of "immediate judge's decisions"- when the German Constitutional Court decided that MR in psychiatric patients lasting longer than 30 minutes requires a judge's immediate decision.	-	-	-	All freedom-restricting devices, e.g. belts in beds, bedrails, movement-restricting blankets, tables attached to a chair.	5% (4989) secluded in 2020. 1% forced medication.	97761 cases in 2020.	4% (4134) restrained in 2020.	Information not provided.	Proportion of patients subjected to MR decreased from 5% in 2017 to 4% in 2019 (p = 0¢000). At the same time, the percentage of patients subjected to seclusion increased from 2.9% to 3.3%.<br>Median duration of MR, seclusion or physical restraint episodes increased by 11% from 6.3 hours to 7.0 hours (p < .001). When looking at these coercive measures individually, only the median duration of seclusion increased statistically significantly.
Guzman-Parra, 2021<br>&<br>Guzman-Parra, 2022	2018	Spain	Study using MR data from all adult acute psychiatric wards of the Andalusian Health Service from July 2016 to December 2018.<br>2021 paper examined impact of a multicomponent intervention to reduce MR; 2022 paper to examine associations with prolonged restraint.	Multicomponent intervention to reduce MR, based on the "Six Core Strategies" 1) leadership, 2) use of data, 3) workforce development, 4) use of restraint prevention tools, 5) consumer roles, 6) debriefing techniques.	Schizophrenia and psychotic disorders 43%, bipolar disorders 24%, personality disorders 8%, substance use 7%, other 18%.<br>Most of the episodes involved patients with schizophrenia or related disorders (42.67%) and bipolar disorders (23.89%).	Mean 42 (SD 14).	Mixed, male 66%	Application of homologated mechanical fastening devices in beds to limit physical mobility.	No seclusion rooms. No regional registers for pharmacological restraint.	17332 people admitted over study period	2567 people restrained (15%), during 3348 admissions that involved restraint (13%).	Last resort when all other measures have been ineffective and the safety of the patient, other individuals or the hospital environment is compromised.<br>In study applied mainly for agitation (52%) and aggression (21%), 7% for self-injury and 11% for other therapeutic activity.	Significant reduction in restraint hours (by 33%), and restraint episodes (by 6%), and proportion of patients restrained (by 8%) over study. Significant decreasing trend in total MR hours during implementation of the intervention, but not in the number of MR episodes.<br>Median MR duration 9.5h (IQR = 5–17). Repeated episodes of prolonged MR, 2–3 episodes: 23%; 4–5 episodes: 5%; and >5 episodes 5%.<br>Wide variability in prolonged restraint between units, range 17% - 81% of episodes. Prolonged restraint associated with time since admission (aOR 0.995), male gender (aOR 1.61), diagnosis (aOR for substance use 1.55), reason for restraint (aOR for agitation 1.44) and the shift on which it was initiated (aOR 3.16 for evening shift).
Guzman-Parra, 2015	2012	Spain	Study of restraint an on acute psychiatric ward in 2005 and 2012, before and after the introduction of a new regulatory protocol designed to reduce the use of restraint.	Protocol which advocates the implementation of standard procedures and monitoring.	Psychotic disorders 35%, affective disorders 26%, substance disorders 10%, anxiety disorders 7%, personality disorders 8%, other 7%.	Mean 43 (SD 13).	Mixed, male 59%.	Fastening devices to limit physical mobility.	-	544 patients admitted (in 2012)	82 (15%) restrained.	To prevent damage to the patient, other people, and/or the physical environment.	MR rate per year was reduced, not significantly, from 18% to 15%. The average duration of each MR episode was significantly reduced from 27.91h to 15.33h. Being female was associated with restraint (aOR 2.32, 95% CI 1.0-5.4).
Harpoth, 2022	2019	Denmark	Retrospective naturalistic pre-post study of all admissions to a university hospital and four regional hospitals, in periods before and after the university hospital relocated to examine for any impact on restrictive practice. Included up to 19% forensic admissions.	Relocation of hospital site from a 170-year-old building to a new purpose built unit.	University hospital: psychotic disorder 35%, mood disorder 24%, personality disorder 11%, substance use 4%.<br>Regional hospitals: Psychotic disorder 28%, mood disorder 22%, personality disorder 11%, substance use 8%>	University hospital: mean 37 (SD 16).<br>Regional hospital: Mean 40 (SD 17).	Mixed, male 45% (university), 42% (regional).	A belt applied around the waist and sometimes straps for the extremities to fix the patient to a bed.	Forced acute medication in 24% of university hospital patients and 27% regional hospital patients.	3519 admissions to university hospital, 6479 to regional hospitals (post-relocation period).	373 MRs in university hospital sample, 345 in regional hospital sample (post-relocation period), not reported as prevalence of total sample.	-	For both samples, MR and involuntary acute medication were aligned and the post-relocation slopes did not deviate significantly from the observed trend before the location.
Hvidhjelm, 2022	2017	Denmark	Stepped-wedge cluster-randomised trial of implementing the short-term assessment of risk and treatability (START). Included all male forensic patients who displayed at least one aggressive episode over the study period across 9 inpatient units.	Use of the short-term assessment of risk and treatability (START).	F20-29 78%, primary or secondary diagnosis of substance use in 64%.	<27, 28%; 28-35, 29%; 36-45, 24%; >45, 19%.	All male.	Restraining straps, belts or other equipment to reduce movement.	-	Total population 156 (94 in control, 42 in START group).	In control group: mean 0.18 episodes of MR (SD 0.4), mean duration 609 minutes (SD 1950).<br>In START group: mean 0.03 episodes (SD 0.1), 274 minutes (SD 1652)	-	The rate of MR use within the START period was 82% lower than that outside the START period (relative risk [RR] 0.18, 95% CI 0.08-0.41). The duration of MR use was 99% lower.

Lickiewicz, 2021	2019	Poland	Comparative study examining 8-month periods before and after introduction of Safewards in a single 50 patient inpatient unit.	Safewards model, which focuses on primary prevention in non-threatening situations, with ten techniques to reduce likelihood of conflict.	Alcohol and drug issues, 37% to 47% at any time, schizophrenia (16%-29%). mood disorders (4%-16%). The remainder were a combination of approximately 50 diagnoses.	Information not provided.	All male.	MRs refer to immobilizing by use of belts or other mechanical means.	-	-	-	Information not provided.	There was a significant difference in the number of patients mechanically re-strained with and without Safewards in place (P< .001). Also reductions in number of episodes, means, time (statistics not reported).<br><br>There was a statistically significant difference between the number of MRs during the day shift (P< .002), nightshift (P< .001), and in the mean for both (P< .001).
Odgaard, 2018	2015	Denmark	Register-based retrospective cohort study of adult inpatients admitted to four wards for affective disorders 2012-2015, with symptoms of mania/hypomania with or without psychosis (excluded first time mania).	Use of the Danish assessment tool for psychiatric inpatients diagnosed with mania (MAS-M).	ICD: F31.0 20.2%; F31.1 35.8%; F31.2 44.0%.	In those not scored with MAS-M, mean 48, in those scored mean 43.	Mixed, male 45% (not scored with MAS-M), 55% (scored with MAS-M).	Restraining to a bed, without the patient's consent, by belt around waist and/or straps around wrists and ankles.	-	218	35 restrained in first week of admission (16%), of whom 17 (49%) belt only, 18 (51%) belt and straps).	In Denmark only if patient exposes self/others to immediate bodily harm or danger to health, harasses or in other ways molests other patients, or commits vandalism of a considerable extent.	Use of MAS-M was not significantly associated with the use of MR.
Perez-Revuelta, 2021	2014	Spain	Retrospective analysis of MR records on an acute mental health unit over an 8-year period (2007-2014). Also compared with period 2000-2007 to examine impact of organisational measures designed to minimise use.	Measures included allowing family to accompany patients during admission process, restraint protocol for registration of staff involved, sociodemographic, clinical and other variables, and reduction of maximum restraint time before psychiatrist review.	Bipolar disorder 15%, personality disorder 15%, psychosis 50%, other 17%.	Mean 42 (SD 13).	Mixed, male 61%.	Wristbands, anklets, belts with magnetic closures and restraint bands to restrict physical mobility.	-	2448 individual patients were admitted 3318 times.	412 admissions (12%) involved MR.	The most common indications for MR were agitation (63%) and/or risk of self-harm (58%), or aggression to others (65%).	Significant reduction in total hours of restraint after the application of the first two organizational changes. Mean hours per admission in 2000-2002 was 12.41 (SD = 4.41). After the second organizational change in 2007, the mean hours 1.27 (SD = 0.51).<br><br>In most cases (75%), restraint was performed within the first 72 h of admission.<br><br>In logistic regression, involuntary admission (OR 6.37; 95% CI 4.82-8.40), and diagnosis of personality disorder (OR 4.71; 3.54-6.78) were significantly associated with MR.
Smith, 2015	2010	United States	Prospective study of the use of seclusion and restraint in Pennsylvanian forensic centres from 2001-2010 (characterised by various measures to decrease the use of containment).	Multiple measures under the "six core strategies" framework (see Guzman-Parra, 2021).	-	-	-	Leather or soft Velcro ankle and wrist restraints.	Rate of seclusion declined from .89 to .04 episodes per 1,000 days (p,.001). Nonsignificant decline in the use of physical restraint.	4805 unique individuals served by centres over study period.	-	-	Peak use of MR was 2002, 1.7 times per 1,000 days, for a total of 372 hours. By 2010, its use significantly declined to .04 episodes per 1,000 days, total of two hours (p,.001). Mean minutes in restraint declined significantly, from 191 minutes per event in 2002 to 39 minutes in 2010 (p <0.01).<br><br>During this decade, rate of patient-to-staff assaults declined, and patient-to-patient assaults was unaffected.
Smith, 2023	2020	United States	Prospective study examining the effects of ending the use of seclusion and MR in six civil hospitals and two forensic centres in Pennsylvania from 2011 to 2020.	Use of seclusion was ended in July 2013 and the use of MR in September 2015.	-	-	-	MR in two orfour body locations with soft Velcro-type restraints.	-	-	-	-	In civil hospitals, MR was applied 118 times for a total of 16,611 minutes, last used in September 2015. In the forensic centers, MR was applied 10 times, involving seven different people for a total of 432 minutes, last applied in 2014.<br><br>Incidents of assault, aggression, and self-injurious behavior significantly declined or were unchanged by the decreasing use of containment procedures.
Stensgaard, 2018	2017	Denmark	Register-based study using interrupted time series analysis to examine whether introduction of Safewards model (start of 2015) reduced coercive measures in adult psychiatric inpatient units, 2012-2017.	Safewards model.	-	43 (IQR 29-55).	Male 53%.	Straps (arms and legs) described separately, MR as a whole not defined.	Of coercive measures in post-intervention period, 4% were forced treatment, 40% forced sedation.	In the post-intervention period 584.7 coercive measures per year, of which 18% MR, 16% straps.	-	-	Rate of MR before implementation of Safewards already significantly decreasing by 4% per quarter (p < 0.001) and was stable in the period after the implementation. No significant difference in rates of commenced MR pre/post implementation.

Stoll, 2022	2020	Switzerland	Pre-post pilot of introduction of monthly moral case deliberation (MCD) on two psychiatric wards.	Moral case deliberation, in which staff meet to reflect collaboratively and systematically on a clinical case.	- (Data at ward / restraint / staff level)	-	-	Tying to a bed.	17% and 10% secluded pre- and post-intervention. 5% and 4% coerced medication pre- and post-intervention.	-	3% of patients were subject to MR before intervention, 2% after intervention.	Information not provided.	Proportions of patients subjected to MR and coerced medication were numerically lower after implementation of MCD without reaching significance. Intensity of MR (summing the duration of individual episodes in the measurement period for each patient concerned) decreased, large effect size; 86.8±45.3 vs. 14.5±12.1 h, exact p=.019, r=-.74).

Supplement 7

Quality of studies reporting impact of an intervention on mechanical restraint

Studies were assessed using the Newcastle-Ottawa scale, and converted to AHRQ (Agency for Healthcare Research and Quality) standards for an overall rating of good quality (3 or 4 stars in selection domain AND 1 or 2 stars in comparability domain AND 2 or 3 stars in outcome/exposure domain), fair quality (2 stars in selection domain AND 1 or 2 stars in comparability domain AND 2 or 3 stars in outcome/exposure domain) or poor quality (0 or 1 star in selection domain OR 0 stars in comparability domain OR 0 or 1 stars in outcome/exposure domain).

Study	Selection (4)	Comparability (2)	Outcome (3)	Total (9)	Overall
An, 2016	○ ● ● ●	● ●	● ● ●	8	Good
Andersen, 2017	● ○ ● ●	○ ○	● ● ●	6	Poor
Badouin, 2023	● ● ● ●	● ●	● ● ●	9	Good
Celofiga, 2022	● ● ● ●	● ●	● ● ●	9	Good
Flammer, 2021<br>&<br>Flammer, 2022<br>&<br>Flammer, 2021b	● ● ● ●	○ ○	● ● ●	7	Poor
Guzman-Parra, 2021<br>&<br>Guzman-Parra, 2022	● ○ ● ●	○ ○	○ ● ●	5	Poor
Guzman-Parra, 2015	● ● ● ●	● ●	● ● ●	9	Good
Harpoth, 2022	● ● ● ●	● ●	● ● ●	9	Good
Hvidhjelm, 2022	○ ● ● ●	● ●	● ● ●	8	Good
Lickiewicz, 2021	● ○ ● ●	● ○	● ● ●	7	Good
Odgaard, 2018	● ○ ● ●	● ●	● ● ●	8	Good
Perez-Revuelta, 2021	● ○ ● ●	○ ○	● ● ●	6	Poor
Smith, 2015	○ ○ ● ●	○ ○	● ● ●	5	Poor
Smith, 2023	○ ○ ● ●	○ ○	● ● ●	5	Poor
Stensgaard, 2018	● ○ ● ●	● ○	● ● ●	7	Good
Stoll, 2022	● ○ ● ●	● ○	● ● ●	7	Good

Supplement 8

Qualitative studies of mechanical restraint.

Study Ref	Study period (last year data collected)	Country	Study details, population, setting	Diagnoses	Age	Sex	Restraint device	Total N of population examined	Indications for MR	Summary of themes and findings
Aluh, 2022	2022	Nigeria	Stable current inpatients at two major neuropsychiatric hospitals. A range of coercive measures were examined but MR themes presented separately. Thematic analysis with a general inductive approach to data coding.	Schizophrenia 40%, mental/behavioural disorder secondary to psychoactive substance 33%, bipolar disorder 10%, depression 10%, schizoaffective disorder 7%.	Mean 35 (SD 10).	Mixed, male 63%.	Chains.	30 participated in focus groups.	Mostly in response to resistance to chemical restraint, and to prevent absconding. Some perceived that was used punitively.	MR was a recurring theme across both study sites (40%, n=12), subtheme entitled "chained like an animal".<br><br>Participants perceived as wrong, dehumanizing, abnormal, humiliating and painful. Also discussed physical injuries from chains and a bladder infection perceived to have been as a result of restriction of movement.
Chien, 2005	2003	China (Hong Kong)	Psychiatric inpatients with violent behaviours recruited from two acute admission wards in a mental health hospital. Participants had been restrained sometime during 2 days prior to the interview, but were stable enough to participate. Data collected via individual interviews, analysis undertaken with theme matching and condensing as per Miles and Huberman.	Schizophrenia 47%, bipolar disorder 20%, psychosis NOS 20%, substance/alcohol abuse 17%, psychopathic personality disorder 10%, schizoaffective 7%, conduct disorder 7%.	Mean 31 (SD 6).	Mixed, 60% male.	Devices designed to confine a patient's bodily movements, such as bilateral limb holders, safety vests, and triangular bandages.	30	Control of aggression and violent behaviours, prevention of injury to patient and staff, prevention of disturbance to other people, maintenance of safety during confusion state.	20 participants voiced positive feelings, 16 negative experiences/frustrations.<br><br>Positive/therapeutic aspects: perception of security and protection, caring behaviour of staff, explanation and frequent interactions, being respected.<br><br>Negative impacts: Perceived lack of concern/empathy, failure to provide information, powerlessness and uncertainty.
Gildberg, 2015	2012	Denmark	Study used documentary analysis of medical records to investigate prolonged MR on a forensic unit, defined as episodes longer than median length identified via the central electronic reporting system.	Of prolonged restraint cases 66% F20-F25, 9% undetermined, 9% F60.8/60.2, 52% F10-19.	Mean 38.8 (of prolonged restraint cases).	22/23 prolonged restraint cases male	Devices such as a belt attached to patient with aim to restrict movement.	23 cases of prolonged restraint were selected, made up 18,382 hours of MR out of 20,476 hours of MR spread across 45 patients.	52% substance misuse prior to restraint. Initial reasons for conflict leading to restraint: behaviour 13%, substance abuse 21%, treatment dropout 65%. Physical violence in 39% before restraint.<br><br>Reasons for prolonged restraint: Protest behaviour 91%, paradoxical behaviour 26%, illness-related behaviour 82%.	Thematic analysis described prolonged episodes characterized by multiple factors—confounding, risk, and alliance parameter—woven into a MR-spiral: patients experience restraint as punishment, injustice, an attack on their person, or torture, react with frustration, reflected in protest behaviours such as anger, violence, verbal aggression, or other forms of behaviour that, from a staff point of view, can be characterized as risk of violence behaviour.
Walker, 2020	2019	Scotland	Study to examine the experience of staff in a high security hospital who were involved in nursing a single patient using a Soft Restraint Kit over a sustained period of time. Study used thematic analysis of data from semi-structured individual interviews.	Patient presented with florid psychotic symptoms.	-	-	Soft restraint kit.	10 clinicians	Patient was unresponsive to medication and was unmanageable within the main ward. Level of violence to self and others extreme and of sufficient intensity that decision to use MR. The patient was isolated from the main ward and nursed in the Modified Strong Room. Initially three belts were used with soft cuffs, one on the chest, abdomen and knees. The patient was lying in a supine position on the floor. The belts were on 24 hours a day. Gradually, one by one the belts were loosened then removed entirely.	Themes (subthemes):<br><br>Sense of responsibility (changes over time, compassion, patient safety, staff safety, dignity, maintaining hope), Aptitude (observation skills physical skills, relational skills [maintaining therapeutic relationship]), inhibitors and enablers (restricted movement, acts of violence, patient's mental state, feeling valued), Consequence (withdrawal, intensity, pressure, anxiety, burnout).

Supplementary references

1. Gildberg FA, Fristed P, Makransky G, Moeller EH, Nielsen LD, Bradley SK. As time goes by: reasons and characteristics of prolonged episodes of mechanical restraint in forensic psychiatry. J Forensic Nurs. 2015;11(1):41-50.
2. Walker H, Tulloch L. A "Necessary Evil": Staff Perspectives of Soft Restraint Kit Use in a High-Security Hospital. Front Psychiatr. 2020;11:357.
3. Hvidhjelm J, Brandt-Christensen M, Delcomyn C, Mollerhoj J, Siersma V, Bak J. Effects of Implementing the Short-Term Assessment of Risk and Treatability for mechanical restraint in a forensic male population: a stepped-wedge, cluster-randomized design. Front Psychiatr. 2022;13:822295.
4. Smith GM, Altenor A, Altenor RJ, Davis RH, Steinmetz W, Adair DK, et al. Effects of ending the use of seclusion and mechanical restraint in the Pennsylvania State Hospital system, 2011-2020. Psychiatr Serv. 2023;74(2):173-81.
5. Smith GM, Ashbridge DM, Altenor A, Steinmetz W, Davis RH, Mader P, et al. Relationship between seclusion and restraint reduction and assaults in Pennsylvania's forensic services centers: 2001-2010. Psychiatr Serv. 2015;66(12):1326-32.
6. Flammer E, Frank U, Steinert T. Freedom restrictive coercive measures in forensic psychiatry. Front Psychiatr. 2020;11:146.
7. Lau S, Brackmann N, Mokros A, Habermeyer E. Aims to reduce coercive measures in forensic inpatient treatment: A 9-year observational study. Front Psychiatr. 2020;11:8.
8. van Heesch B, Boucke J, De Somer J, Dekkers I, Jacob E, Jeandarme I. The use of coercive measures in a high security setting in Belgium: Prevalence and risk factors. Int J Law Psychiatry. 2022;82:101792.
9. Harpoth A, Kennedy H, Terkildsen MD, Norremark B, Carlsen AH, Sorensen LU. Do improved structural surroundings reduce restrictive practices in psychiatry? Int J Ment Health Syst. 2022;16(1):53.
10. Andersen K, Nielsen B. Coercion in psychiatry: the importance of extramural factors. Nord J Psychiatry. 2016;70(8):606-10.
11. Noorthoorn E, Lepping P, Janssen W, Hoogendoorn A, Nijman H, Widdershoven G, et al. One-year incidence and prevalence of seclusion: Dutch findings in an international perspective. Soc Psychiatry Psychiatr Epidemiol. 2015;50(12):1857-69.
12. Birkeland S. Threats and violence in the lead-up to psychiatric mechanical restraint-a Danish complaints audit. Journal of Forensic Psychiatry and Psychology. 2018;29(1):99-105.
13. Leerbeck SM, Mainz J, Boggild H. Use of coercion at Danish psychiatric wards by day of week and time of day. Dan Med J. 2017;64(8).
14. Linkhorst T, Birkeland SF, Gildberg FA, Mainz J, Torp-Pedersen C, Boggild H. Use of the least intrusive coercion at Danish psychiatric wards: A register-based cohort study of 131,632 first and subsequent coercive episodes within 35,812 admissions. Int J Law Psychiatry. 2022;85:101838.
15. Martensson S, Johansen KS, Hjorthoj C. Dual diagnosis and mechanical restraint - a register based study of 31,793 patients and 6562 episodes of mechanical restraint in the Capital region of Denmark from 2010-2014. Nord J Psychiatry. 2019;73(3):169-77.
16. Eguchi R, Onozuka D, Ikeda K, Kuroda K, Ieiri I, Hagihara A. Psychological assessment of acute schizophrenia patients who experienced seclusion either alone or in combination with restraint. Int J Psychiatry Med. 2018;53(3):171-88.
17. Di Lorenzo R, Baraldi S, Ferrara M, Mimmi S, Rigatelli M. Physical restraints in an Italian psychiatric ward: clinical reasons and staff organization problems. Perspect Psychiatr Care. 2012;48(2):95-107.

18. Wu WW. Psychosocial correlates of patients being physically restrained within the first 7 days in an acute psychiatric admission ward: retrospective case record review. East Asian Arch Psychiatry. 2015;25(2):47-57.
19. Chien WT, Chan CW, Lam LW, Kam CW. Psychiatric inpatients' perceptions of positive and negative aspects of physical restraint. Patient Educ Couns. 2005;59(1):80-6.
20. Flammer E, Eisele F, Hirsch S, Steinert T. Increase in coercive measures in psychiatric hospitals in Germany during the COVID-19 pandemic. PLoS ONE. 2022;17(8):e0264046.
21. Flammer E, Hirsch S, Steinert T. Effect of the introduction of immediate judge's decisions in 2018 on the use of coercive measures in psychiatric hospitals in Germany: a population-based study. Lancet Reg Health Eur. 2021;11:100233.
22. Staggs VS. Trends in Use of Seclusion and Restraint in Response to Injurious Assault in Psychiatric Units in U.S. Hospitals, 2007-2013. Psychiatr Serv. 2015;66(12):1369-72.
23. Hirose N, Morita K, Nakamura M, Fushimi K, Yasunaga H. Association between the duration of physical restraint and pulmonary embolism in psychiatric patients: A nested case-control study using a Japanese nationwide database. Arch Psychiatr Nurs. 2021;35(5):534-40.
24. Knutzen M, Bjorkly S, Eidhammer G, Lorentzen S, Helen Mjosund N, Opjordsmoen S, et al. Mechanical and pharmacological restraints in acute psychiatric wards--why and how are they used? Psychiatry Res. 2013;209(1):91-7.
25. Knutzen M, Bjorkly S, Eidhammer G, Lorentzen S, Mjosund NH, Opjordsmoen S, et al. Characteristics of patients frequently subjected to pharmacological and mechanical restraint--a register study in three Norwegian acute psychiatric wards. Psychiatry Res. 2014;215(1):127-33.
26. Knutzen M, Sandvik L, Hauff E, Opjordsmoen S, Friis S. Association between patients' gender, age and immigrant background and use of restraint--a 2-year retrospective study at a department of emergency psychiatry. Nord J Psychiatry. 2007;61(3):201-6.
27. Kostecka M, Zardecka M. The use of physical restraints in Polish psychiatric hospitals in 1989 and 1996. Psychiatr Serv. 1999;50(12):1637-8.
28. Valimaki M, Lam YTJ, Hipp K, Cheng PYI, Ng T, Ip G, et al. Physical Restraint Events in Psychiatric Hospitals in Hong Kong: A Cohort Register Study. Int J Environ Res Public Health. 2022;19(10):16.
29. Aluh DO, Ayilara O, Onu JU, Grigaite U, Pedrosa B, Santos-Dias M, et al. Experiences and perceptions of coercive practices in mental health care among service users in Nigeria: a qualitative study. Int J Ment Health Syst. 2022;16(1):54.
30. Agbor VN, Takah NF, Aminde LN. Prevalence and factors associated with medication adherence among patients with hypertension in sub-Saharan Africa: protocol for a systematic review and meta-analysis. BMJ Open. 2018;8(3):e020715.
31. Hoy D, Brooks P, Woolf A, Blyth F, March L, Bain C, et al. Assessing risk of bias in prevalence studies: modification of an existing tool and evidence of interrater agreement. J Clin Epidemiol. 2012;65(9):934-9.
32. Odgaard AS, Kragh M, Roj Larsen E. The impact of modified mania assessment scale (MAS-M) implementation on the use of mechanical restraint in psychiatric units. N J Psychiatry. 2018;72(8):549-55.
